# Exploring Cuba’s population structure and demographic history using genome-wide data

**DOI:** 10.1038/s41598-018-29851-3

**Published:** 2018-07-30

**Authors:** Cesar Fortes-Lima, Jonas Bybjerg-Grauholm, Lilia Caridad Marin-Padrón, Enrique Javier Gomez-Cabezas, Marie Bækvad-Hansen, Christine Søholm Hansen, Phuong Le, David Michael Hougaard, Paul Verdu, Ole Mors, Esteban J. Parra, Beatriz Marcheco-Teruel

**Affiliations:** 1 UMR7206 Eco-Anthropology and Ethno-Biology, CNRS-MNHN-University Paris Diderot, Musée de l’Homme, Paris, 75016 France; 2 Department for Congenital Disorders, Statens Serum Institut, Copenhagen, 2300 Denmark; 3 The Lundbeck Foundation Initiative for Integrative Psychiatric Research, iPSYCH, Aarhus University, Aarhus, 8000 Denmark; 4 National Centre of Medical Genetics, Medical University of Havana, Havana, 10600 Cuba; 5 Centre for Sociological and Psychological Research, Havana, 10600 Cuba; 6 Department of Anthropology, University of Toronto, Mississauga, ON L5L 1C6 Canada; 7 Psychosis Research Unit, Aarhus University Hospital, Risskov, Aarhus, 8240 Denmark

## Abstract

Cuba is the most populated country in the Caribbean and has a rich and heterogeneous genetic heritage. Here, we take advantage of dense genomic data from 860 Cuban individuals to reconstruct the genetic structure and ancestral origins of this population. We found distinct admixture patterns between and within the Cuban provinces. Eastern provinces have higher African and Native American ancestry contributions (average 26% and 10%, respectively) than the rest of the Cuban provinces (average 17% and 5%, respectively). Furthermore, in the Eastern Cuban region, we identified more intense sex-specific admixture patterns, strongly biased towards European male and African/Native American female ancestries. Our subcontinental ancestry analyses in Cuba highlight the Iberian population as the best proxy European source population, South American and Mesoamerican populations as the closest Native American ancestral component, and populations from West Central and Central Africa as the best proxy sources of the African ancestral component. Finally, we found complex admixture processes involving two migration pulses from both Native American and African sources. Most of the inferred Native American admixture events happened early during the Cuban colonial period, whereas the African admixture took place during the slave trade and more recently as a probable result of large-scale migrations from Haiti.

## Introduction

The islands of the Caribbean were one of the last regions of the Americas to be colonized by anatomically modern humans^1^. According to archaeological evidence, two groups of hunter-fisher-gatherers associated with different cultures (Ortoiroid and Casimiroid), entered the archipelago at different times. The first group originated from northern South America (Venezuela and Guyana), settled in Trinidad around 6,000 BCE, and then expanded to the north along the Lesser Antilles^1,2^. The second group moved around 4,500–4,000 BCE from the Yucatan Peninsula to Hispaniola and Cuba—the largest island of the Greater Antilles^1,2^. Around 350 BCE, the arrival of Arawak-speaking people from Venezuela, associated with the horticulturalist Saladoid culture, changed the anthropological landscape in the Caribbean, and became the major group^3^. At the time of the first contacts with European settlers, Cuba was inhabited by three autochthonous groups: the “Guanahatabey”; the “Ciboney” or “Western Taíno”; and the “Classic Taíno” (living in Western, Central, and Eastern Cuba, respectively)^1,3^.

Late in the fifteenth century, the arrival of Spanish settlers had a profound impact on the indigenous population of the Caribbean^4^. The indigenous Cuban population was decimated during the early colonial period^5,6^. The Spanish settlers then started to bring sub-Saharan enslaved Africans^7,8^. During the transatlantic slave trade (TAST) from 1526 to 1875, approximately 853,000 enslaved Africans were forcibly deported to Cuba^9,10^. The vast majority (92%) arrived during the last phase of the TAST from 1801 to 1875^10,11^. The most important embarkation regions were West-Central Africa (30.0% embarked from modern Republic of Congo, Democratic Republic of Congo, and Angola), the Bight of Biafra (26.1% from modern Eastern Nigeria, Cameroon, Equatorial Guinea, and Gabon), the Bight of Benin (14.2% from modern Togo, Benin, and Western Nigeria), Southeast Africa (11.1% from modern Southern Tanzania, Mozambique, and Madagascar), Sierra Leone (9.6% from modern Guinea Bissau, Guinea, and Sierra Leone), the Gold Coast (4.0% from modem Ghana), the Senegambia (3.8% from modern Senegal and Gambia), and the Windward Coast (1.2% from modem Liberia and Ivory Coast)^9–11^. While broad embarkation regions can be identified based on historical evidence^9,12^, the geographical origins of enslaved Africans forcibly displaced during the TAST remain unclear.

Throughout the colonial and post-colonial periods, European migrations coming to Cuba primarily originated from different regions of the Iberian Peninsula^6,12^. The nineteenth century witnessed the arrival of over 125,000 contract workers from China, almost exclusively males (99%), to work in the sugar plantations^6,13^. Although archaeologists and historians have reconstructed the migratory processes underlying Cuban demographic history, a number of long-standing questions remain unsolved, mostly due to the patchy archaeological and historical records^6^, and the incomplete and biased information regarding the illegal slave trade^12^.

Previous genetic studies have provided relevant insights into the population history and admixture dynamics in Cuba^14–16^. Based on a limited set of mitochondrial DNA (mtDNA) and Y-chromosome markers from 254 Cuban individuals, Mendizabal *et al*.^14^ highlighted a strong sex-bias in the admixture process, with male lineages mostly tracing to Europe and female lineages to Africa. Cintado *et al*.^15^ employed a small panel of seventeen ancestry informative markers (AIMs) to characterize admixture proportions in 206 individuals from Havana—the largest city in Cuba. Marcheco-Teruel *et al*.^16^ carried out admixture analyses using around 1,000 individuals from all the fifteen Cuban provinces, and a panel of 128 AIMs as well as diagnostic mtDNA and Y-chromosome markers. These authors reported extensive variation in admixture proportions in different Cuban regions. The studies highlighted above were hampered by the relatively small number of genetic markers considered. More recently, Moreno-Estrada *et al*.^17^ used genome-wide microarray data to study a diverse set of Caribbean populations, including 80 individuals from Cuba. They were able to investigate distinct subcontinental source populations. Likewise, other recent studies^18–20^ have employed dense genomic datasets to provide insights into the genetic structure of the Cuban population. However, the sample sizes of these studies were relatively small and did not capture well the demographic composition of the present Cuban population. Additionally, the number of continental reference populations considered in these studies was also quite limited.

Here, we substantially expand our previous research^16^ to explore Cuba’s population structure and demographic history, by using a dense genome-wide SNP dataset of 860 individuals born in Cuba. Importantly, this Cuban sample covers all the fifteen Cuban provinces and is an excellent representation of the current distribution of the Cuban population in terms of sex, age, and population density. Based on both non-parametric and haplotype-based methods, we first describe genetic diversity patterns in the present-day Cuban population in relation to other worldwide populations, including Hispanic/Latino populations in the Americas. The inferred distributions of continental ancestries reflect broad-scale geographic admixture patterns across Cuba, and unravel regional differences within and between Cuban provinces. We then evaluate whether there are differences in patterns of sex-specific admixture in Cuba. To delve deeper into the founding of the Cuban population, we address hypotheses regarding the Cuban putative ancestral source populations by analysing genetic affinities with populations from specific regions in Africa, Europe, and the Americas. Furthermore, we address whether gene flow in the Cuban population has been the result of recent multiway migration events involving different continental sources. We applied a model-testing approach to infer and reconstruct admixture histories across Cuba in the context of the TAST and after its abolition. Overall, our research emphasizes finer-scale aspects of genetic diversity patterns in the current Cuban population, provides new insights on the geographical origins of enslaved Africans forced to move to Cuba, and highlights complex demographic histories during and after the slave trade period.

## Results

### Genetic diversity and dissimilarity patterns across Hispanic/Latino populations

**Figure 1:**
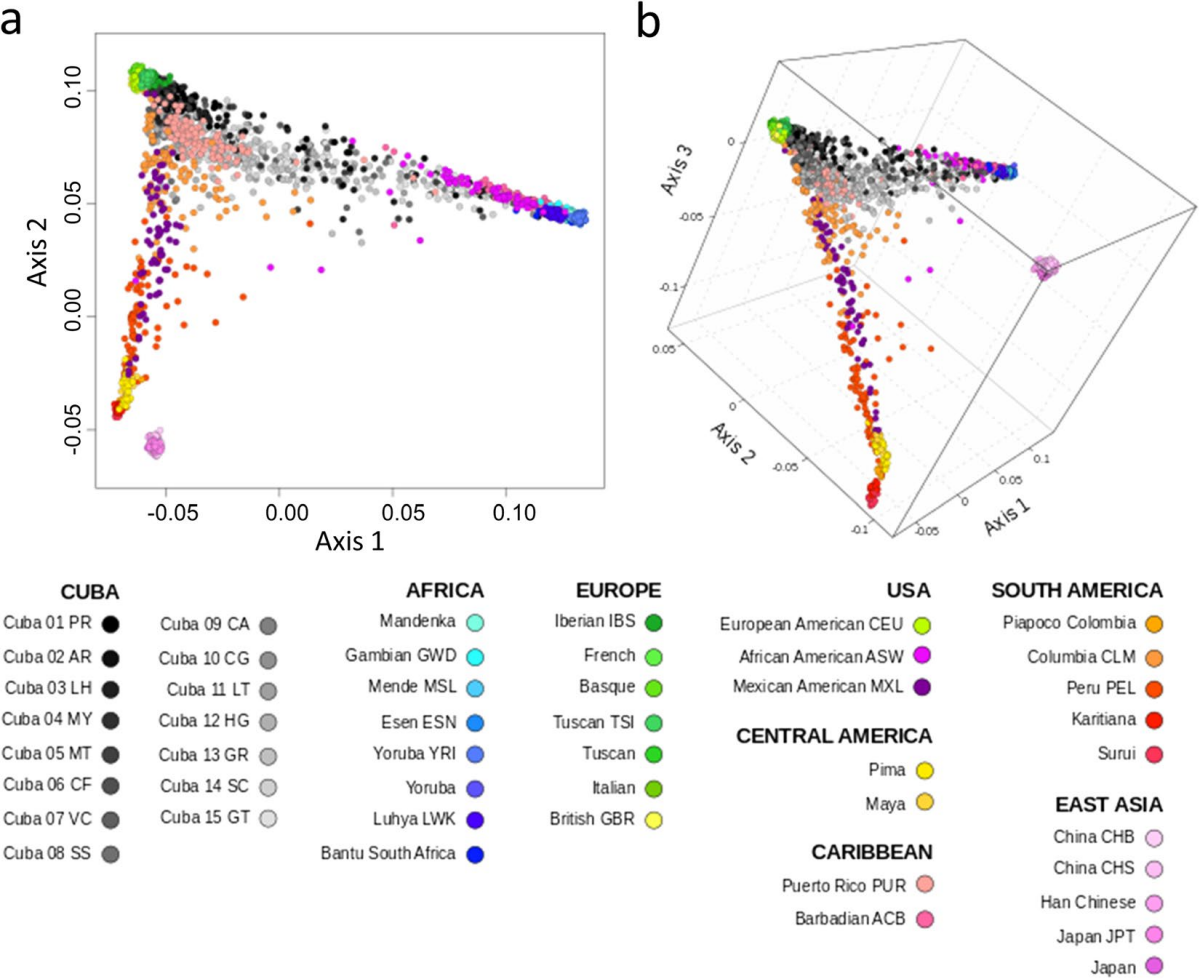
Genetic diversity in Hispanic/Latino populations. MDS plots based on pairwise ASD matrix for the Cuban provinces and worldwide populations included in the called “Cuba-World” dataset (see further details in Supplementary Table S1), using (**a**) two-dimensional and (**b**) three-dimensional metric MDS projections for the first, second, and third axes. Most Cuban individuals are much closer to the European populations than the African populations. This is consistent with the Cuban population having experienced a three-way continental admixture between European, African, and, to a lesser extent, Native American source populations.

To explore inter-individual genetic variation in Cuban and other Hispanic/Latino populations, we first evaluated allele-sharing dissimilarity (ASD)^21^ across all pairs of individuals in the dataset of Cuban and worldwide populations (Supplementary Table S1). The multidimensional scaling (MDS) plot based on the ASD matrix shows highly variable genetic patterns across populations having experienced the TAST (Fig. 1a), including Hispanic/Latino populations in the Americas. These patterns are consistent with three-way admixture from Native American, European, and, to a lesser extent, African source populations^22–24^. The Cuban individuals lie on the European-African trajectory, similarly to African-descendants from USA and Barbados, although most Cuban individuals are much closer to the European cluster than the African cluster (Fig. 1b). These patterns are corroborated by the ancestry inference analyses described below.

We then explored the genetic diversity patterns in the Cuban population in comparison with other worldwide populations, using a genome-wide average haplotype heterozygosity approach that compensates for the known ascertainment bias of SNP chips at the worldwide scale^25,26^. Across Cuban provinces (Supplementary Fig. S1), we found average haplotype heterozygosity values (range: 0.78–0.84) that are intermediate between those of European and African populations (range: 0.74–0.79 and 0.83–0.85, respectively). Within Cuban provinces, higher average haplotype heterozygosities were detected in two Eastern Cuban provinces (Guantanamo: 0.84 and Santiago de Cuba: 0.83) than in the rest of Cuba (on average: 0.80 SD = 0.01). As described below, these two Cuban provinces have higher African ancestry that other Cuban provinces. The average heterozygosity levels are lower in Native American populations, or in the case of Hispanic/Latino populations, in populations with high Native American ancestry proportions. Other Hispanic/Latino populations have haplotype heterozygosity levels intermediate between those of European and Native American populations (range between 0.47 and 0.65). Although the differences in heterozygosity levels between populations might be the result of different factors^25,26^, in general, admixture would be expected to result in the observed levels of heterozygosity that are intermediate between those of the parental populations. Therefore, these results might reflect the variable admixture histories among Hispanic/Latino populations in the Americas that have undergone the TAST^17,27^.

### Population structure in Cuba

**Figure 2:**
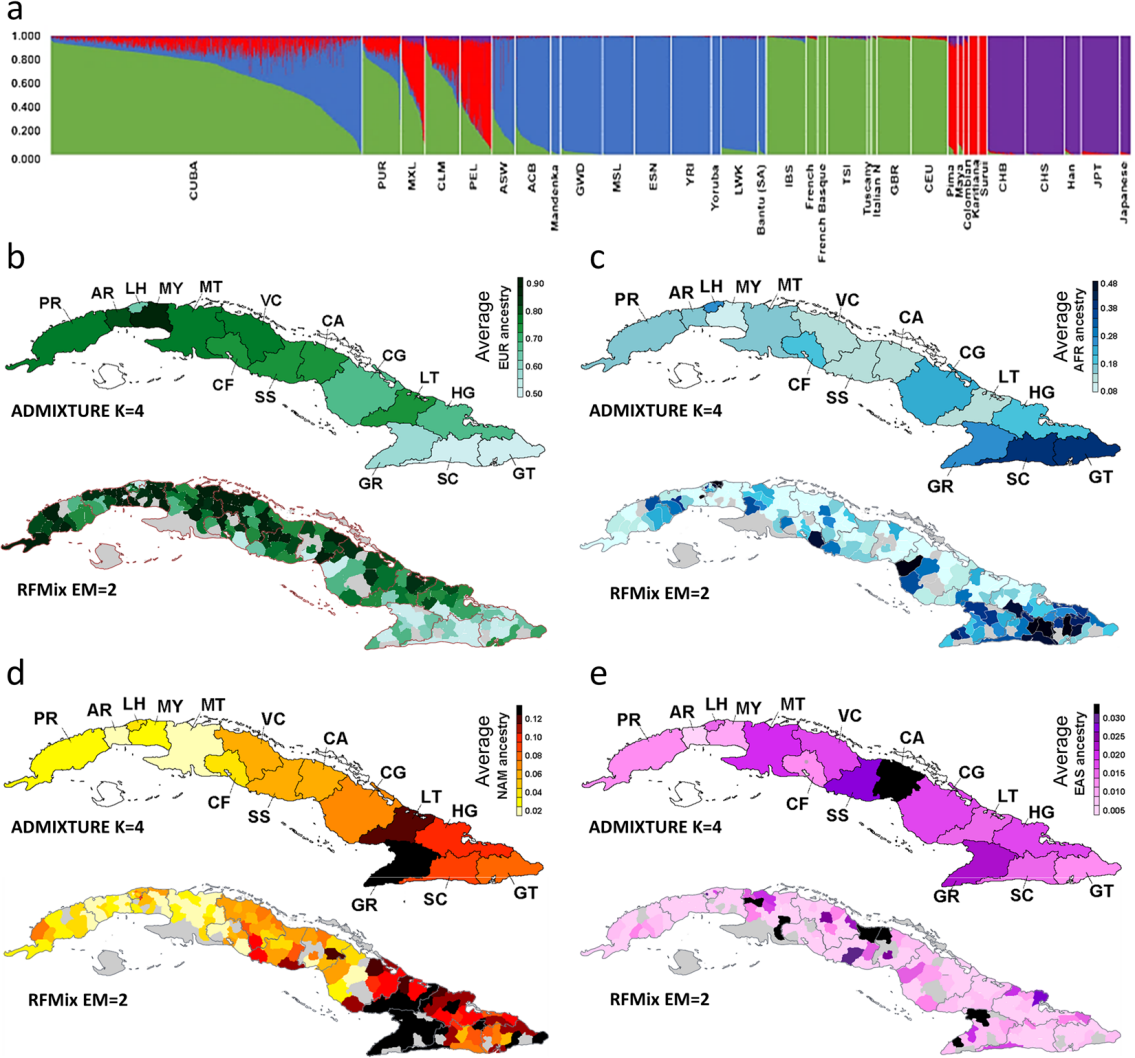
Genetic diversity across Cuba. (**a**) Bar-plot of four-way continental ancestry proportions in Cuban individuals and worldwide individuals estimated using unsupervised ADMIXTURE analysis at K = 4. Geographic distribution of (**b**) European (or green cluster), (**c**) African (or blue cluster), (**d**) Native American (or red cluster), and (**e**) East Asian (or purple cluster) ancestries across Cuban provinces estimated using unsupervised ADMIXTURE analysis at K = 4 (map on the top), and across Cuban municipalities estimated using RFMix analysis (EM = 2) (map on the bottom). Figure showing in grey the Cuban municipalities that were not included in the study (Supplementary Fig. S16a).

To examine genetic diversity patterns within Cuba, we performed both supervised and unsupervised ADMIXTURE analysis at K = 3 and K = 4 (Supplementary Table S2). Each model-based approach includes three and four parental populations based on the known demographic history of the Cuban population, which has been summarized in the Introduction. The results of a supervised ADMIXTURE analysis at K = 3 and K = 4 are in close agreement with those obtained in the unsupervised analysis (see Supplementary Table S2). Figure 2a depicts in a graphical format the results of the unsupervised ADMIXTURE analysis at K = 4. This analysis shows variable admixture patterns across Cuban provinces (Supplementary Fig. S2). On average, the proportion of European ancestry estimated across all Cuban samples was 71% SD = 20.2% (Fig. 2b). The highest European ancestry was observed in Western provinces such as Mayabeque (on average 87.7% SD = 9.6%). In contrast, Fig. 2c shows elevated levels of African admixture among individuals from La Havana (on average 27.9% SD = 27.7% across 72 individuals), and the Eastern provinces of Santiago de Cuba and Guantanamo (on average 38.8% SD = 24.0% across 88 individuals and 40.1% SD = 25.2% across 54 individuals, respectively). Interestingly, Cuban individuals with relatively high Native American ancestry are also located in the Eastern Cuban provinces (Fig. 2d), but in Granma and Las Tunas instead (on average 12.4% SD = 4.4% across 74 individuals and 13.9% SD = 3.7% across 38 individuals, respectively). Finally, no clear geographic pattern is observed for the few individuals with traces of East Asian ancestry (Fig. 2e). Only 37 Cuban individuals (4.3% of the Cuban sample) show proportions of East Asian ancestry higher than 5%, hence evidencing the limited contribution of the East Asian gene-pool to the Cuban population genetic diversity (on average across all Cuban samples: 1.7% SD = 2.5%). Therefore, these results are in general agreement with the genetic diversity results described above (Fig. 1), and could similarly be interpreted as signatures of a predominantly three-way admixture among European, African, and Native American populations at the source of Cuban genetic diversity.

To further estimate the continental ancestry in our dataset, we used the local ancestry calls from the RFMix analysis, calculating global ancestry proportions in our sample on the basis of three- and four-way continental admixture models. The RFMix (EM = 2) results at the individual, municipal, and provincial scales were largely in agreement with the supervised and unsupervised ADMIXTURE results at K = 3 and K = 4 (see Fig. 2 and Supplementary Table S2). As reported in previous studies^17,23,28^, ADMIXTURE and RFMix individual-level admixture estimates are highly correlated (Spearman’s rho: 0.997 European, 0.986 African, 0.986 Native American, and 0.537 East Asian ancestry, all of them are highly significant: *P*-value < 0.001) (Supplementary Fig. S3). Furthermore, the RFMix analysis of the four-way admixture model reveals that Cuban individual haploid genomes present a complex mosaic of continental ancestry tracts (see Supplementary Fig. S4).

To further investigate the ancestry tracts observed in Cuba, we divided the ancestry tracts estimated using RFMix (EM = 2) into two categories: short (between 5 and 50 cM) and long ancestry tracts (>50 cM). For each continental ancestry in each Cuban province, we then performed two types of analyses based on these data (see Supplementary Table S3). First, we compared the proportions of all short tracts that are of each ancestry with the proportions of all long tracts that are of each ancestry. On average across all the fifteen Cuban provinces (Supplementary Fig. S5), we found that the proportion of short ancestry tracts that are of European ancestry is lower than the proportion of long tracts that are of European ancestry (on average 63.4% SD = 14.4% and 80.0% SD = 30.6%, respectively), highlighting once again the strong European ancestry in the present-day Cuba population. In contrast, the proportion of short tracts that are of Native American ancestry is much higher than the proportion of long tracts that are of Native American ancestry (on average 13.6% SD = 7.3% and 0.7% SD = 1.9%, respectively). These differences are not as pronounced in the case of short and long ancestry tracts that are of African (on average 21.6% SD = 15.0% and 18.7% SD = 30.3%, respectively), or East Asian ancestry (on average 1.4% SD = 2.7% and 0.6% SD = 3.6%, respectively). Second, for each ancestry, we compared the proportions of tracts that are short with the proportions of tracts that are long. In agreement with the first analysis, the proportion of European tracts that are short is lower than the proportion of European tracts that are long (on average 41.2% SD = 26.6% and 59.8% SD = 48.0%, respectively). Notably, for the Native American tracts the proportion of tracts that are short is much higher than the proportion of tracts that are long (on average 96.5% SD = 88.1% and 3.5% SD = 11.9%, respectively), suggesting limited recent Native American gene flow in the Cuban population. We further analysed the distribution and frequency of all ancestry tracts to reconstruct the timing of the admixture scenarios that better fit the observed data in Cuba (see below).

We also investigated the distribution of genomic segments that are identical-by-descent (IBD) between pairs of individuals from the Cuban population. This analysis highlights that individuals from the Eastern provinces (e.g. Holguin, Last Tunas, Gramma, and Guantanamo) share more IBD segments than individuals from other Cuban provinces (Supplementary Fig. S6 and Supplementary Table S4). In particular, the average total length of IBD segments shared by pair of individuals within and between Holguin and Las Tunas (18.5 Mb within Holguin, 17.0 Mb within Las Tunas, and 15.6 Mb between Holguin and Las Tunas) is considerably higher than the length shared by pairs of individuals within or between other Cuban provinces (range: 1.6–11.0 Mb within and 0.7–7.5 Mb between). This stands in sharp contrast to the total length of IBD segments shared by individuals from Havana, which is only 1.6 Mb. These provincial differences are also evident in Supplementary Fig. S7, which graphically depicts the average cumulative length of IBD segments shared by pair of individuals within and between the Cuban provinces, and in Supplementary Fig. S8, which represents graphically the length of IBD segments shared by 16 randomly selected individuals from each province.

### Sex-specific admixture patterns in Cuba

**Figure 3:**
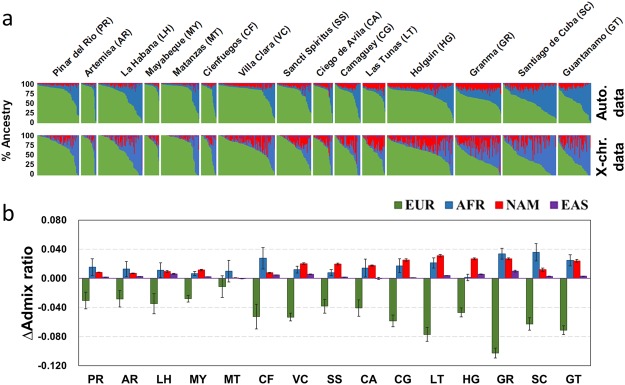
Sex-specific admixture patterns in Cuba. (**a**) Bar-plots of individual ancestry proportions across Cuban provinces estimated using RFMix (EM = 2) on the basis of autosomal (top; Auto.) and X-chromosome (bottom; X-chr.) data. Each vertical line represents one individual based on individual fractions for European (EUR), African (AFR), Native American (NAM), and East Asian (EAS) ancestry. (**b**) ΔAdmix ratios in all the fifteen Cuban provinces show different sex-specific admixture patterns across the island. Bar-plots for each ancestry were plotted with respective confidence intervals (95% CI).

We detected patterns of sex-biased gene flow in all fifteen Cuban provinces (Fig. 3). We found higher African and Native American ancestries on the X-chromosome than on autosomes, and the opposite pattern for European ancestry (Fig. 3a, Supplementary Table S5, and Supplementary Fig. S9). The admixture difference ratios (ΔAdmix ratios)^29^ between the X-chromosome and autosomes show positive values when considering African and Native American ancestries (Fig. 3b), thus reflecting higher female-specific admixture from both continental ancestries, while negative values for European ancestry evidence an excess of European male-specific admixture. Particularly, Eastern provinces have more extreme differences between X-chromosome and autosomal continental ancestries than Western provinces (Wilcoxon signed-rank test *P*-value = 0.0001). For example, the differences estimated based on both sexes for European ancestry are highest in Granma (−17.7%), the differences for African ancestry are highest in Santiago de Cuba (7.8%), and the differences for Native American ancestry are highest in Las Tunas (7.9%) (see first “X-chr-Auto” column in Supplementary Table S5). Those results are in strong agreement with the sex-specific admixture using a modeling framework^30^ (Supplementary Table S5). Overall, these results evidence stronger biases towards European male and African/Native American female ancestries in Eastern provinces than in Western provinces (Fig. 3).

### Subcontinental ancestral origin of Cuban source populations

**Figure 4:**
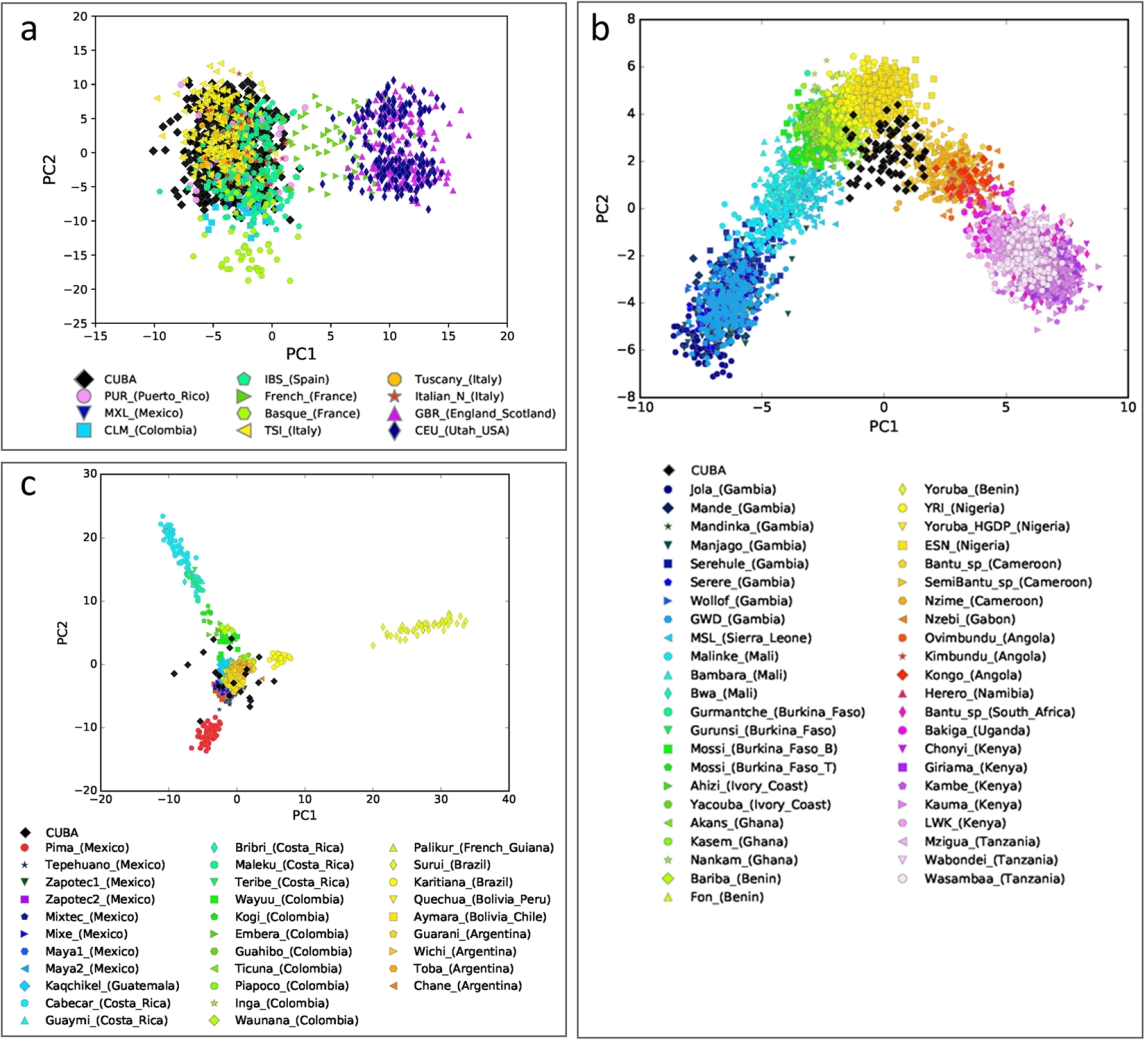
Fine-scale genetic structure across non-masked reference populations and masked Cuban haploid genomes. Figure showing the ancestry-specific MDS obtained for each ancestry using the approach described in Browning *et al*.^18^ (MDS-based ASPCA). The placement of masked Cuban individuals against a backdrop of non-masked reference populations for (**a**) European, (**b**) African, and (**c**) Native American ancestries.

We explored the possible source populations involved in the observed genetic mosaic of Cuban genomes. To do so, we merged our Cuban dataset with available publicly genome-wide SNP datasets for European, African, and Native American ancestry (Supplementary Tables S1, S6 and S7, respectively). Figure 4a shows the fine-scale population structure of European ancestry in Cuba observed with the ancestry-specific MDS approach described in Browning *et al*.^18^ (MDS-based ASPCA). The first principal component (PC1) separates the Southern and Northern European populations, while the PC2 splits the French Basque population from other European populations. In agreement with the known historical sources of European migrations to Cuba^6,9^, the Cuban haplotypes of European ancestry overlap with Southern European populations from Spain and Italy rather than with Northern or Central European populations. This result is supported by analyses indicating that, when comparing the Cuban haplotypes of European ancestry with haplotypes from European populations, the lowest ancestry-specific Weir and Cockerham’s weighted *F*_*ST*_ (WC-*F*_*ST*_) values (0.0037) are observed between the Cuban and Iberian population (Supplementary Table S8). The MDS analysis based on the ASD matrix shows very similar patterns (Supplementary Fig. S10), with the Cuban individuals sharing strong genetic affinities with the cluster associated with Southern European populations.

To investigate the subcontinental African ancestral origin in Cuba, we used, as reference, 45 sub-Saharan African populations (Supplementary Table S6). In the MDS-based ASPCA of African ancestry (Fig. 4b), PC1 separates Western African populations from Southern and Eastern African populations, and PC2 further separates West African populations on a geographical West-to-East pattern going from Gambia to Nigeria and Cameroon. Consistent with historical sources^6,9^, African haplotypes from Cuba primarily cluster with African populations associated with the Bights of Benin and Biafra, as well as West-Central Africa. Similar trends were observed in the MDS plot based on the ASD matrix (Supplementary Fig. S11), and the analysis of the length of IBD segments shared between pairs of Cuban and African individuals (Supplementary Fig. S12). Additionally, these trends are also supported by the WC-*F*_*ST*_ analysis. The lowest ancestry-specific WC-*F*_*ST*_ values were observed between the Cuban population and African populations from the aforementioned regions (Supplementary Table S8).

For the subcontinental Native American ancestral origin, we analysed masked Cuban haploid genomes together with haploid genomes from 31 Native American populations (Supplementary Table S7). In the MDS-based ASPCA of Native American ancestry (Fig. 4c), the Surui from Brazil, Cabecar from Costa Rica, and Pima from Mexico are the three major outliers observed among Native American populations, as previously described^17,31,32^. The Cuban haplotypes of Native American ancestry present a wide overlap with individuals from the remaining Native American populations. The IBD analysis primarily points to a Native American ancestral origin mainly associated with South American populations (Supplementary Fig. S13). These findings were supported by the ancestry-specific WC-*F*_*ST*_ values (range: 0.33–0.40) estimated between the Cuban Native American haplotypes and Native American haplotypes from different regions in South America (Supplementary Table S8). We also increased the number of markers included on the analyses to 244,227 SNPs (Supplementary Methods), after merging the Cuban dataset with available whole-genome data of worldwide populations included in the Simons Genome Diversity Project (hereafter; SGDP)^33^. Although the SGDP has a limited number of Native American samples from Latin America (22 samples in total; see Supplementary Table S9), the MDS-based ASPCA and ancestry-specific WC-*F*_*ST*_ values focused on Native American ancestry of Cuban samples are in close agreement with our previous findings (Supplementary Fig. S14 and Supplementary Table S9).

### Admixture times and demographic migrations

**Figure 5:**
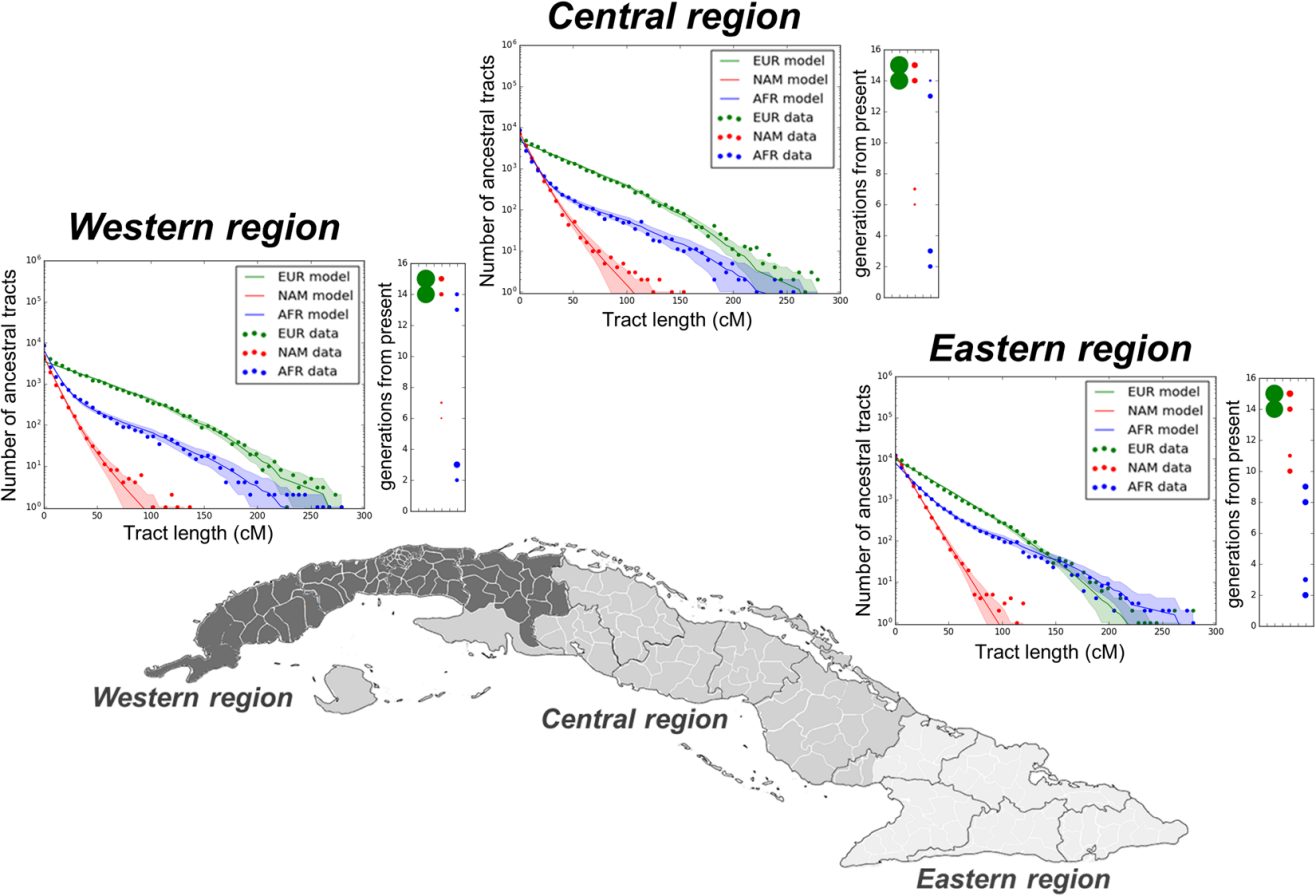
Time-frame of admixture events estimated in Cuba using TRACTS analysis. Figure showing the best-fitting model based on length distributions of ancestry tracts within each Cuban region, allowing for one European, two African, and two Native American migration events. The data points represent the observed distribution of ancestry tracts estimated using RFMix (EM = 2), while solid-coloured lines represent the distribution from the predicted model, and shaded areas indicating confidence intervals (68.3% CI) of the predicted model. The admixture timing of the best-fitting model is displayed to the right of each graph. Sizes of the dots represent inferred average proportions for each ancestry (see Supplementary Table S10). Best-fitting models were selected based on the strength of the log-likelihood of each Cuban region (−566.7 in Western, −553.2 in Central, and −505.8 in Eastern region).

To reconstruct the recent admixture histories in Cuba, we tested five admixture scenarios with one (or two) migration pulse(s) for European, Native American, and African ancestries (Supplementary Fig. S15). For each scenario, Cuban individuals were pooled into the three historical regions (Western, Central, and Eastern departments) that were used for political and administrative purposes in Cuba from 1827 to 1878. Subsequent subdivisions of these major regions gave rise to the current Cuban political-administrative distribution^34^. In all three historical regions in Cuba, a model with one migration pulse for European and two major pulses of migration for both African and Native American ancestries best fits the tract-length distribution of our data (Fig. 5). In the Western and Central Cuban regions, our results evidence early admixture dates for both the African and Native American initial pulses (14–15 generations ago), while the second pulses were estimated to occur much later (3–4 and 7–8 generations ago, respectively) (Supplementary Table S10). In the Eastern Cuban region, the second Native American pulse is older (11–12 generations ago) and more intensive (9%) than in the rest of Cuba (range 1–2%), in agreement with a more intensive gene flow from indigenous groups^35^. In this region, the first African pulse was more recent (dating 9–10 generations ago) and more intense (17%) than in the rest of Cuba (range 6–7%) (Supplementary Table S10). In the three Cuban regions, the second African pulse was very recent (at 3–4 generations ago; 1805–1834 CE), evidencing recent African gene flow after the abolition of the slave trade in Cuba in 1820^34^. These findings point to different demographic histories among the three major historical regions in Cuba, in agreement with the results obtained with other analytical methods described above. It is important to note that we only considered five plausible admixture models in Cuba, and there may be other models that were not explored that may provide a better fit to the observed data. However, current model-testing approaches to infer admixture times do not allow testing of more complex admixture scenarios that may better reflect the history of admixture in the Cuban population^36–39^.

## Discussion

Cuba has a complex history of population migrations^6,7^, with notable differences compared to neighbouring Hispanic/Latino populations in the Americas (Fig. 1). Our study explores fine-scale aspects of the genetic diversity present in both within and between Cuban provinces (Fig. 2b–e). We found admixture patterns highly structured geographically across the island, with the Eastern provinces having higher African and Native American ancestry components than the rest of the Cuban provinces (Supplementary Table S2). The admixture proportions estimated using RFMix analysis are highly correlated with those obtained in a previous study^16^ including the same samples and based on a panel of 128 AIMs (r = 0.98 for European ancestry; r = 0.97 for African ancestry; and r = 0.96 for Native American ancestry). Thus, supporting that a carefully selected AIM panel is an efficient tool to capture continental admixture proportions^40^.

In agreement with Marcheco-Teruel *et al*.^16^, the observed geographic admixture patterns in Cuba are fully consistent with archaeological and historical sources^35,41^. Estimates dating back to 1510 indicate that most of the indigenous groups in Cuba were located in the Eastern part of Cuba^42^, in which the most populated areas correspond to the present-day Holguin and Granma provinces, and where there is the highest concentration of Taíno archaeological sites^41–43^. Currently, both Eastern provinces have the highest Native American ancestry in Cuba (Fig. 2d and Supplementary Table S2). Further, the highest African admixture proportions observed in municipalities within Havana and Santiago de Cuba provinces are consistent with these regions being, respectively, the first and second major slave ports in Cuba during the TAST^35^.

Our analyses focusing on patterns of IBD sharing show denser intra- and inter-provincial connections in the Eastern region than in the Western Cuban region (Supplementary Figs S7 and S8). In particular, the extent of IBD sharing is much higher within and between Holguin and Las Tunas than within or between any other provinces (Supplementary Table S4 and Supplementary Fig. S6). This finding of higher shared recent common ancestry in Eastern Cuba, and more particularly, Holguin and Las Tunas, is in agreement with the higher prevalence of Mendelian diseases that has been reported in Eastern provinces such as Holguin^44,45^, where endogamy was a common practice during the colonial period^46^. Conversely, Havana shows the lowest average (1.55 Mb/pair) of the shared IBD between pairs of individuals (Supplementary Table S4). This reflects that Havana, as the most important metropolitan area in the country^47^, has attracted considerable migration from other Cuban provinces resulting in lower levels of IBD sharing.

Historical sources point to a marked gender imbalance during the TAST^48,49^, with more enslaved African men than women forcefully brought to Cuba (on average 69%)^10,11^, and more Spanish men than women migrating to that region (81% of Spanish immigrants)^6,7^. However, Marcheco-Teruel *et al*.^16^ showed that the Native American and African ancestry proportions in Cuba were higher in maternally-inherited mtDNA haplogroups (35% and 39%, respectively) than in autosomal AIMs (8% and 20%, respectively), and in turn, the autosomal AIMs estimates were higher than the paternally-inherited Y-chromosome haplogroups (0.5% and 18%, respectively). Based on much denser autosomal and X-chromosome SNP data, we also find significant African/Native American contributions from the maternal side and European contributions from the paternal side in the current Cuban population (Fig. 4, Supplementary Fig. S9, and Supplementary Table S5). In particular, ΔAdmix ratios show more pronounced sex-specific admixture patterns in Eastern provinces than in Western provinces.

It is important to note that our findings in Cuba do not necessarily extrapolate to all Hispanic/Latino populations, in particular for African ancestry. In agreement with our study, Conomos *et al*.^19^ reported higher maternal African ancestry in a Cuban and Dominican sample, and similar African autosomal and X-chromosome contributions in a sample from Puerto Rico. However, in their Mexican, Central American, and South American samples, estimates of African X-chromosome admixture proportions were lower than the autosomal estimates, supporting higher African paternal ancestry in these groups. Bryc *et al*.^27^ described an increased paternal African ancestry in Mexico, Colombia, Ecuador, and Dominican Republic, but similar autosomal and X-chromosome ancestral proportions in Puerto Rico. Rishishwar *et al*.^29^ reported an excess of maternal Native American ancestry and paternal African ancestry in Mexico, Ecuador, Dominican Republic, and Colombia. However, in Puerto Rico they reported an excess of maternal African ancestry, as we observe in Cuba. Therefore, Hispanic/Latino populations have different patterns of admixture as well as sex-specific admixture, underlining the notable heterogeneity in social dynamics and colonial past among those admixed populations during the TAST and after its abolition.

Historical records indicate that the European migrations to Cuba came mainly from Spain^9,12^. One of the most relevant Spanish migration took place between 1882 and 1930, when 3.5 million people left Spain for Cuba^50^. In agreement, our results point to a Cuban European ancestral component deriving primarily from Southern European populations (Fig. 4a)^17^. Further, based on historical data obtained from over 2,500 slave vessels that arrived in Cuba during the TAST^9,10^, West-Central Africa and the Bights of Biafra and Benin regions were the three major source population of enslaved Africans (in total 70%)^6,10^. Our results indicate that the African ancestral component of Cuban individuals falls between Benin, Nigeria, Cameroon, Gabon, and Angola (Fig. 4b), supporting those three African historical regions as the best proxy for the forced transcontinental African migration in the Cuban population.

The identification of the Native American ancestral component in the Cuban population is extremely challenging, mainly due to the lack of modern or ancient genome-wide SNP data of non-admixed indigenous groups from the Circum-Caribbean region^17,33,51,52^; the limited number of markers available for comparative purposes; the overall low Native American genetic contribution to the current Cuban population; and the strong effect that genetic drift has had on some indigenous groups in the Americas^31,53^. Our MDS-based ASPCA does not provide clear evidence about the region of origin of Native American haplotypes in the Cuban genomes (Fig. 4c and Supplementary Fig. S14). However, the ancestry specific WC-*F*_*ST*_ and IBD analyses evidence genetic links between Cuban individuals and several Native American populations from South America and Mesoamerica (Supplementary Fig. S13 and Supplementary Table S8).

Historical sources describe a strong presence of enslaved Africans in the Western and Central Cuban regions during the TAST, closely related to the development of the sugar plantation economy, which barely moved farther eastward in the island^7,34^. From the late sixteenth century onward, enslaved Africans were primarily concentrated in Western Cuba, specifically within and around the port city of Havana^34^. Over 350,000 enslaved Africans disembarked in La Havana from 1551 to 1875, representing 41.1% of the slave population arrived in Cuba^10^. Consistent with historical sources evidencing the early arrival of enslaved Africans in Western and Central Cuba during the TAST^6,9,10^, the first African migration pulse was inferred at fifteen generations ago (1502 CE, 95% CI:1486–1515) in these two regions, which is notably earlier than in the Eastern region (1631–1660 CE) (Fig. 5 and Supplementary Table S10).

According to historical sources^6,9,10^, the slave trade in Eastern Cuba was less intensive and delayed compared to the rest of Cuba. For instance, in Santiago de Cuba, over 55,000 enslaved Africans disembarked from 1701 to 1875 (6.5% of the slave population in Cuba)^10^. However, our estimated ancestry fractions highlight stronger African ancestry and haplotype heterozygosities in the Eastern Cuban provinces such as Santiago de Cuba and Guantanamo (Fig. 2c and Supplementary Fig. S1). Furthermore, these provinces have higher frequency of long African ancestry tracts (on average 41%) than the other Cuban provinces (14%) (Supplementary Fig. S5), suggesting a strong recent African gene flow in Eastern Cuba. This is consistent with intracontinental migrations in the Caribbean, and more particularly, with the recent large-scale migration from Haiti to Cuba^35,54^. The Haitian population has one of the highest averages (84%) of African ancestry in the Caribbean^17^. Between 1913 and 1931, legal migration from Haiti to Cuba was estimated to be over 189,000 migrants, and illegal migration further brought in 450,000–600,000 migrants^55^. Our admixture dates (3–4 generations ago) evidence a recent second African migration pulse in Cuba that is especially high (15%) in the Eastern region, consistent with the expected impact of these Haitian large-scale migrations.

For the Native American ancestry, the first migration pulse occurred with a high intensity early during the colonial period, around 15 generations ago (Fig. 5 and Supplementary Table S10). This is also supported by higher proportions of short tracts than long ancestry tracts of Native American ancestry in Cuba (Supplementary Fig. S5). It is widely believed among historians that the indigenous Cuban populations were extinct by the seventeenth century^1,6^. Nevertheless, this perspective has been challenged in recent studies^43,56^, which report the presence of indigenous communities living in Cuba long after the end of the seventeenth century. Our results support this latter perspective, since we evidenced a Native American genetic admixture pulse in the post-contact Cuban population form Western and Central Cuba, albeit of small absolute magnitude, between 7 and 8 generations ago.

In conclusion, Cuba has experienced major demographic migrations involving multiple indigenous groups, European settlers, and enslaved Africans, as well as recent intracontinental large-scale population movements in the Americas. The current Cuban population has different genetic patterns of admixture than other Hispanic/Latino populations, and there is also evidence of population structure across the island. European ancestry is higher in Western than in Eastern provinces, except for La Havana. Conversely, Eastern provinces have higher Native American and African ancestries, though there is also evidence of strong genetic differentiation within this region (e.g. higher Native American ancestry in Granma, Holguin and Las Tunas, and higher African ancestry in Guantanamo and Santiago de Cuba). Therefore, the extensive genetic structure observed within and between Cuban regions emphasizes the need to ensure appropriate representation of ancestrally diverse individuals in future biomedical and genetic association studies in Cuba^20,57^. For the African gene-flow, our subcontinental ancestry analyses point to populations from West-Central Africa and the Bights of Benin and Biafra regions as the major source populations of the African ancestral gene pool in Cuba. In addition, our results provide evidence of a strong and recent second African migration pulse in Eastern Cuba, most probably reflecting recent Haitian large-scale migrations. Overall, these new findings expand our understanding of the impact of demographic events associated with mass migrations and population admixture in Cuba, as well as the ancestral origins of the Cuban source populations, with unprecedented geographic resolution.

## Methods

### Data collection and genotyping procedure

The original sample size comprised 1,019 voluntary participants born in Cuba representing all fifteen Cuban provinces (Supplementary Fig. S16). These samples constitute an excellent representation of the current distribution of the Cuban population in terms of sex, age, and population density (Supplementary Methods). A detailed comparison of the relative proportions of each category in the studied Cuban sample and the Cuban census^47^ was published elsewhere^16^.

A total of 957 DNA samples were genotyped using the Infinium PsychArray v1.0 and v1.1 BeadChips (Illumina Inc.) at Statens Serum Institut, Denmark (http://www.ssi.dk/english.aspx) (Supplementary Methods). During the genome-wide quality control (QC) procedure, 97 samples were removed due to their high missing call rate (i.e. >1%). After some basic filtering based on Hardy-Weinberg (p < 10^−6^) and minor allele frequency (i.e. <1%), we created an initial dataset that includes 432,138 polymorphic autosomal SNP markers. We estimated genetic relatedness for all pairs of individuals using KING^58^ and PC-Relate^59^, and no first-degree or second-degree relatives were found (Supplementary Fig. S17). After additional QC procedures using PLINK v1.90^60^ (see Supplementary Methods), the remaining linkage disequilibrium (LD-) unpruned dataset includes 292,549 SNPs genotyped in 860 unrelated Cuban individuals (range between 24 and 108 individuals per Cuban province). In addition, we assembled another LD-unpruned dataset only for X-chromosome SNP markers that includes 5,060 SNPs genotyped in 860 Cuban individuals.

### Population structure analyses

To better understand the genetic contribution from each continental source population to the genetic landscape of present-day Cuba, we merged our Cuban dataset with other datasets comprising 1,353 unrelated individuals from continental reference populations in Europe, Africa, America, and Asia (hereafter-called “Cuba-World” dataset, see Supplementary Table S1 and Supplementary Fig. S18)^22,26^. For the LD-unpruned Cuba-World dataset, we first used *asd* v1.0 (https://github.com/szpiech/asd) to compute ASD between all pairs of individuals separately^21^. We represent the respective inter-individual pairwise ASD distance matrix into two- or three-dimensional metric MDS projections using the *cmdscale* function in R^25^. We then estimated genome-wide heterozygosities for the Cuba-World dataset. To avoid SNP-chip geographical ascertainment bias when calculating population SNP-by-SNP heterozygosities, we employed a haplotype heterozygosity approach following Verdu *et al*.^25^, which was derived from the method developed by Li *et al*.^26^, and using the recombination map from the HapMap Phase 2 project Build GRCh37/hg19.

To estimate three- and four-way continental ancestry for each individual, we first used the clustering algorithm implemented in the software ADMIXTURE v1.30^61^. For the LD-pruned Cuba-World dataset, we performed both supervised and unsupervised ADMIXTURE analysis at K = 3 and K = 4. We then conducted the local ancestry deconvolution approach at the within-individual genomic level using RFMix v1.5.4^62^, based on the autosomal haploid genomes in the LD-unpruned Cuba-World dataset (see Supplementary Methods). We first used SHAPEIT2^63^ to generate haplotypic phased data for each assembled dataset, and then we ran RFMix in “PopPhased” mode using two steps of Expectation-Maximization algorithm (EM = 2)^62^. We collapsed inferred ancestry calls to calculate the average ancestry proportions for each individual haploid genome, Cuban municipality, and Cuban province. We focused on average ancestry proportions across all, short (between >5 and ≤50 cM), and long (>50 cM) ancestry tracts^17,28^.

To investigate the distribution of genomic IBD segments shared between pairs of individuals from the Cuba-World dataset, we use the Refined-IBD^64^ tool implemented in the software BEAGLE v4.1^65^. The minimum length threshold was set to 3 cM^66^. We then estimated the total length of IBD segments (in Mb) shared IBD between pairs of individuals from the same province and different provinces in Cuba.

### Estimating sex-biased gene flow

To explore if there is evidence of sex-specific admixture patterns in Cuba, we estimated admixture proportions in the X-chromosome dataset using RFMix (EM = 2), and compared them to the estimates based on autosomal data using three approaches. First, we applied the method proposed by Rishishwar *et al*.^29^ to calculate the ΔAdmix ratio. We expect ΔAdmix ratios to be positive when there is an excess of female-specific admixture contributions from one specific ancestry; or negative when there is an excess of male-specific admixture instead^29^. Second, we applied Wilcoxon signed-rank two-sided unpaired test to assess the difference between the paired X-chromosome and autosomal ancestry proportions of individuals from each Cuban province^17^. Third, we estimated sex-specific admixture under the sex-specific admixture mechanistic modelling framework developed in (p. 271 in^30^), with a fixed number of 15 generations^30,67^.

### Inferring subcontinental ancestry

To investigate the ancestral origins of the Cuban population, we used haplotype-based methods for individual Cuban haploid genomes masked for one specific ancestry. To do so, we merged our Cuban dataset with publicly available whole-genome and genome-wide SNP datasets for European, African, and Native American ancestries (called “Cuba-World”, “Cuba-Africa”, and “Cuba-America” SNP dataset, respectively) (see Supplementary Fig. S17 and Supplementary Tables S1, S6, and S7), and we then inferred genetic links between the Cuban population and putative ancestral populations included in each reference population panel using three genome-wide methods. First, we used *asd* v1.0 to compute ASD between all pairs of individuals separately for each assembled SNP dataset and visualize them in the MDS based on ASD, as described above. Second, we analysed ancestry calls estimated for each haploid individual in the RFMix analysis, to generate new datasets with non-masked reference haplotypes and masked Cuban haplotypes with different thresholds for each continental ancestry (i.e. 75%, 60%, 50%, 40%, 30%, and 15%). The only exception was the analysis of Native American ancestry, in which we only used a 15% cutoff due to the fact that only one Cuban sample has more than 30% Native American ancestry. For each dataset, we used the MDS approach described in Browning *et al*.^18^, to perform MDS-based ASPCA for within-continental ancestry inference^18,28^. We found better overlapping for non-masked reference haplotypes and masked Cuban haplotypes with at least 40% of European ancestry, or 75% of African ancestry, or 15% of Native American ancestry, and plots from those thresholds were included in the manuscript (see Fig. 4). Moreover, for the Native American ancestry we repeated the analysis using a dataset with an increased number of SNPs, but lower number of Native American populations. After merging the Cuban dataset with 40 worldwide populations included in the SGDP, the QC-filtered LD-unpruned dataset included 244,227 SNPs (called “Cuba-SGDP” SNP dataset) (see Supplementary Table S9). We then estimated genetic affinities between pairwise populations based on ancestry-specific WC-*F*_*ST*_^28,68^ using VCFTools^69^. Third, we estimated the total length of IBD segments (in Mb) shared between Cuban individuals and individuals from different European, African and Native American populations.

### Modelling demographic history and admixture timing

To explore the recent migration history in Cuba, we applied the model-testing approach implemented in the TRACTS software^37^. This analysis allows fitting tract-length distributions expected under predefined complex admixture scenarios to all the observed tract-length distributions in our Cuban dataset using RFMix (EM = 2), to infer the admixture parameters (timing and intensity of admixture events) underlying the observed data^37^. We first pooled Cuban individuals into the three historical regions (Western, Central, and Eastern departments) that have administratively structured Cuba from 1827 to 1878^34^. For each historical department, we then tested five admixture models with different admixture pulses. We started the first model with a founding admixture event between European and indigenous groups fifteen generations ago followed by a pulse of African admixture (1^st^EUR,1^st^NAM +1^st^AFR). Separately, we evaluated models adding an additional: African pulse (1^st^EUR, 1^st^NAM +1^st^AFR +2^nd^AFR), Native American pulse (1^st^EUR, 1^st^NAM +2^nd^NAM +1^st^AFR); Native American and African pulse (1^st^EUR, 1^st^NAM +2^nd^NAM +1^st^AFR +2^nd^AFR), and Native American, African, and European pulse (1^st^EUR, 1^st^NAM +2^nd^NAM +1^st^AFR +2^nd^AFR +2^nd^EUR). To identify the best-fitting model, we first fit each model with 1,000 starting parameter randomizations, and then evaluated the magnitude of log-likelihood of each competing model^17,24^. Finally, we calculated the dates of the admixture event assuming 29 years per generation^70^.

### Data availability statement

The data that support the findings of this study are available from the Cuban Government but restrictions apply to the availability of these data, which were used under license for the current study, and so are not publicly available. Data are however available from the authors upon reasonable request and with permission of the Cuban Government. For accessing the data, within Cuban legal framework, contact the Cuban Centre of Medical Genetics (CNGM) Research Ethics Committee at Medical University of Havana (cngm@infomed.sld.cu). Requests should be addressed to Dr. Hilda Roblejo, vice-president of the CNGM Research Ethics Committee (hilda.roblejo@infomed.sld.cu).

### Ethical statement

This study was conducted following the ethical principles for medical research included in the World Medical Association Declaration of Helsinki, and was approved by the CNGM Research Ethics Committee at Medical University of Havana, Cuba. Collected human DNA samples were obtained in Cuba, and each voluntary participant gave written informed consent prior to the private interview, physical examination, and blood sample collection.

## Electronic supplementary material

Supplementary Methods

All supplementary Tables and supplementary Figures

Supplementary Tables S1-S10 in Excel
