# Supplementary material for: Exploring Cuba’s population structure and demographic history using genome-wide data
Source: Sci Rep. 2018 Jul 30;8:11422. doi: 10.1038/s41598-018-29851-3 (PMC6065444; doi:10.1038/s41598-018-29851-3)
Supplement: Supplementary file 1 — Supplementary Methods [file 41598_2018_29851_MOESM1_ESM.docx]

**Exploring Cuba’s population structure and demographic history using genome-wide data**

**Cesar Fortes-Lima^1,+^, Jonas Bybjerg-Grauholm^2^, Lilia Caridad Marin-Padrón^3^, Enrique Javier Gomez-Cabezas^4^, Marie Bækvad-Hansen^2^, Christine Søholm Hansen^2^, Phuong Le^5^, David Michael Hougaard^2^, Paul Verdu^1^, Ole Mors^6,7,8,9^, Esteban J. Parra^5,^*^,+^ Beatriz Marcheco-Teruel^3,^***

# Supplementary Methods S1

## **Genotyping procedure**

The original sample size comprised 1,019 individuals representing all fifteen Cuban provinces. Participants were selected from 1,229 households located in 137 of the 168 Cuban municipalities using the Kish method[^1^](https://paperpile.com/c/78IEi6/OnREd) for each household, to ensure that all the members of the household had the same probability of being selected for the study. The selection procedure was made in collaboration with the National Statistics Office from Cuba, to select individuals on the basis of demographic characteristics of the Cuban provinces and municipalities in terms of sex, age, population density, and rural/urban residence[^2^](https://paperpile.com/c/78IEi6/Ngzpu). To avoid batch biases, DNA samples were randomized before being processed at Statens Serum Institut in Copenhagen, Denmark (<http://www.ssi.dk/english.aspx>). Due to problems with DNA quality, twelve samples were excluded from the genotyping. The samples were genotyped using the Infinium PsychChip v1.0 and v1.1 arrays (Illumina Inc., San Diego, California, USA), in accordance with the manufacturer's instructions. Both arrays are designed for genotyping 593,260 SNPs, of which 312,905 SNPs are common (~53% of total), 282,097 SNPs are exonic markers (~48% of total), 167,352 SNPs are intronic markers (~28% of total), 106,285 SNPs are in low-frequency (~18% of total), and 141,172 SNPs are rare (~24% of total) based on weighted allele frequencies in the samples of the 1000 Genomes Project. Additionally, the chip includes 56,113 SNPs for biomedical research that were not included in the analyses.

Expected clustering positions were trained in both arrays using the cluster algorithm GenTrain2 included in GenomeStudio version v2011.1 (Illumina Inc., San Diego, California, USA), and DNA strand positions were built according to the Genome Reference Consortium Human genome build 37 (GRCh37/hg19). Following the autoclustering method, positions were manually curated if they had a call frequency below 90% in both autosomal and sex chromosomes, and had GenTrain scores below 0.5 and/or cluster separation below 0.2[^3^](https://paperpile.com/c/78IEi6/kzKgF). The call sets were exported from GenomeStudio using PLINK Input Report Plug-in v2.1.1. After exporting mapping positions, identifiers were updated based on updated BLAST information for the probe sequences (Broad Institute, Boston, USA), before being merged using PLINK v1.90[^4^](https://paperpile.com/c/78IEi6/zqNy0). This procedure and the hierarchy of mappings chosen is described in Pedersen et al[^5^](https://paperpile.com/c/78IEi6/gFWu4).

## **Quality control procedure**

The initial dataset comprised 957 samples and 586,967 markers. Prior to the statistical analyses, we performed the following quality control (QC) steps^6^ to filter out samples and/or markers.

Based on sample-QC steps, we removed: 97 samples with missing call rates >1%; duplicates based on pi-hat values; heterozygosity outliers (het >5xSD); samples with conflicts between the sex inferred using the heterozygosity-rate of the X-chromosome and the sex indicated in the Cuban registry, or with evidence of genotyping problems based on X-chromosome or Y-chromosome markers (e.g. putative males with many heterozygous X-chromosome markers); and one of the pair of individuals with potential cryptic relatedness (pi-hat values >0.2).

Based on marker-QC steps, we removed: non-autosomal SNPs (570,038 markers remaining), non-biallelic SNPs, markers showing strong deviations from Hardy-Weinberg (*P*<10^-6^, 567,022 markers remaining); markers with missing call rates >5% (562,501 markers remaining); monomorphic SNPs (432,138 markers); low frequency SNPs (MAF<1%, 318,522 markers remaining); indels (318,069 markers remaining); markers with strand conflicts with 1000 Genomes Project Phase 3 (1KGP) dataset[^6^](https://paperpile.com/c/78IEi6/do7vN), and large frequency differences (>20%) with the 1KGP Hispanic/Latino samples (300,595 markers remaining); markers that were reported to have problems with rs-Id numbers, and/or chromosome and genomic locations during a re-blast of the Psychchip probes.

After-QC steps, the final number of markers was 292,549 markers in 860 individuals. We performed KING[^7^](https://paperpile.com/c/78IEi6/gNuH) and GENESIS[^8^](https://paperpile.com/c/78IEi6/9HiM) to estimate genetic relatedness and IBD sharing probabilities for pair of individuals. We used the iterative procedure implemented in GENESIS to estimate both PC-AiR and PC-Relate that can infer the population structure and recent genetic relatedness in our dataset, respectively. After two iterations of PC-AiR and PC-Relate, we did not detect individuals with high relatedness probabilities included in the dataset (Supplementary Fig. S17).

In addition, we assembled another dataset including only X-chromosome SNP markers. Additional QC steps were implemented for this dataset, we removed: markers for which there were more than 10 males with heterozygous calls; markers with strand conflicts with 1KGP dataset, such as large frequency differences (>20%) with the 1KGP Hispanic/Latino samples; markers with missing call rates >1% and minor allele frequencies <1%; and markers with Hardy-Weinberg *P*-values<10^-4^ in females. The final number of X-chromosome markers was 5,060 SNPs in 860 Cuban individuals.

## **Assembled genome-wide SNP datasets**

To carry out a diverse set of genome-wide analyses, we merged the Cuban data with publicly available datasets of worldwide populations, assembling three datasets with different SNP genotyping densities (Supplementary Fig. S18). We first merged the Cuban data with 1,349 continental reference samples included in the 1KGP[^6^](https://paperpile.com/c/78IEi6/do7vN) and the Human Genome Diversity Project (HGDP) samples genotyped in the Illumina’s HuHap 650K array[^9^](https://paperpile.com/c/78IEi6/0Tmz6) (Supplementary Table S1). The resulting dataset was called the “Cuba-World” dataset (Cuba, 1KGP, and HGDP datasets), which includes 143,136 autosomal markers for 2,213 individuals (LD-unpruned Cuba-World dataset), and 43,359 SNPs after pruning for linkage disequilibrium (LD) (LD-pruned Cuba-World dataset). For the LD-pruning process, we removed one SNP from a pair of SNPs if the LD was greater than the threshold *r^2^*= 0.1, for SNPs within a 50-SNP sliding window and advanced by ten SNPs each time. We then performed the same QC-steps to obtain the X-chromosome Cuba-World dataset, which includes 2,420 SNPs for 2,213 individuals.

For analyses focused on subcontinental ancestral contributions, we further increased the reference population samples in order to obtain a broader geographical representation of within-continental African and Native American populations. In the case of African populations, we combined the Cuba-World dataset with genotype data available for 45 sub-Saharan African populations[^10–15^](https://paperpile.com/c/78IEi6/IpTYq+8Hwaz+iN1ki+53izP+urKqi+W7FLR) (Supplementary Table S6). After merging and QC, the resulting dataset was called “Cuba-Africa” dataset, and included 42,910 SNPs in 3,859 people for the LD-unpruned dataset, and 28,421 SNPs after LD-pruning. Likewise, in the case of Native American populations, we combined the Cuba-World dataset with genotype data of 31 Native American populations from the Americas presented by Reich et al,[^16^](https://paperpile.com/c/78IEi6/g5N5O/?noauthor=1) (Supplementary Table S7). After merging and QC, the resulting dataset was called “Cuba-America” dataset, and included 22,388 SNPs in 2,616 people for the LD-unpruned dataset, and 16,024 SNPs after LD-pruning.

To further investigate the Native American ancestry in the Cuban population, we increase the number of SNPs using whole-genome data of populations included in the Simons Genome Diversity Project (hereafter; SGDP)[^17^](https://paperpile.com/c/78IEi6/Twdmy). In total 22 Native American samples from 10 populations across Latin America are included in the SGDP dataset (Supplementary Table S9). To avoid size biases resulting from unbalanced reference panel sizes in the analyses, we therefore used 22 samples in each continental reference panel. We therefore merged the Cuban samples with 88 samples included in the SGDP, the resulting dataset was called ‘Cuba-SGDP’ (Supplementary Table S9). After QC, this dataset included 244,227 SNPs in 41 populations

## **Phasing procedure and local ancestry deconvolution**

For local ancestry deconvolution analyses, we first used SHAPEIT2[^18^](https://paperpile.com/c/78IEi6/xBY0K) to generate haplotypic phased data for each assembled dataset. To maximize phasing accuracy, the dataset was phased using as a reference panel the 1000 Genomes Phase 3 reference panel of haplotypes, and as a genetic map the human reference genetic map from HapMap Phase II b37[^19^](https://paperpile.com/c/78IEi6/t5iCF). Before phasing, we used SHAPEIT2 in check mode to exclude SNPS with Mendelian inconsistencies. For the X-chromosome dataset, we used SHAPEIT2 in X-chromosome phasing mode[^18^](https://paperpile.com/c/78IEi6/xBY0K), and we coded admixed males as homozygous individuals by paring the haploid X-chromosome of each individual to avoid inconsistencies in haploid males[^20^](https://paperpile.com/c/78IEi6/mwDtj).

We then applied the random-forest algorithm of RFMix to infer the putative ancestry for small chromosomal segments. The input to RFMix consisted of Cuban phased genotype data and each set of reference panels representing four continental ancestries. To avoid sample-size bias among the reference panels, we randomly selected for each reference panel 94 non-admixed individuals (i.e. we included in the analysis the 94 reference samples with >95% for that ancestry, as estimated using ADMIXTURE analysis at K=4). We excluded the first and the last 2Mb from the telomeres of each chromosome, due to observed inaccurate LAI in those regions[^21^](https://paperpile.com/c/78IEi6/F4Q4w). We ran RFMix in PopPhased mode to estimate local ancestry likelihoods in 0.2 cM genetic windows, with “generations after admixture” parameter set to fourteen generations[^22^](https://paperpile.com/c/78IEi6/lDqPF), and other parameters were left as their default arguments[^23^](https://paperpile.com/c/78IEi6/Qqde8). We performed two steps of machine learning using two steps of Expectation-Maximization algorithm (EM=2), to iteratively improve local ancestry calls at each chromosomal region[^23^](https://paperpile.com/c/78IEi6/Qqde8). Local ancestry assignments were determined using a 0.9 posterior probability threshold for each genetic window[^24^](https://paperpile.com/c/78IEi6/ldod). We then collapsed inferred ancestry calls for each chromosomal region to calculate the average local ancestry proportions for each Cuban individual haploid genome, each Cuban municipality, and each Cuban province. Moreover, we calculated the average ancestry proportions across short (between >5 and ≤50 cM) and long (>50 cM) ancestry tracts, following recommendations from previous studies[^25,26^](https://paperpile.com/c/78IEi6/ReEd+JHKs). For the X-chromosome dataset, we coded admixed males as homozygous individuals by paring the haploid X-chromosome of each individual, and we paired reference males into pseudo-diploid individuals within each continental reference panel to avoid making haploid reference males look more frequent than they really are[^20^](https://paperpile.com/c/78IEi6/mwDtj).

To better visualize the geographic distribution of admixture patterns in the Cuban population, we used the R package *maptools*[*^27^*](https://paperpile.com/c/78IEi6/VgFue) to compile spatial distribution maps for estimated ancestral proportions in the ADMIXTURE and RFMix analyses. Furthermore, we plotted the haplotypic ancestry calls across autosomal chromosomes for the individual for each Cuban province with similar average admixture proportions than in the corresponding province.

# Supplementary References

1. [Kish, L. *Survey Sampling*. (John Wiley and Sons, 1965).](http://paperpile.com/b/78IEi6/OnREd)

2. [Marcheco-Teruel, B. *et al.* Cuba: exploring the history of admixture and the genetic basis of pigmentation using autosomal and uniparental markers. *PLoS Genet.* **10,** e1004488 (2014).](http://paperpile.com/b/78IEi6/Ngzpu)

3. [Zhao, S. *et al.* Strategies for processing and quality control of Illumina genotyping arrays. *Brief. Bioinform.* (2017). doi:](http://paperpile.com/b/78IEi6/kzKgF)[10.1093/bib/bbx012](http://dx.doi.org/10.1093/bib/bbx012)

4. [Chang, C. C. *et al.* Second-generation PLINK: rising to the challenge of larger and richer datasets. *Gigascience* **4,** 7 (2015).](http://paperpile.com/b/78IEi6/zqNy0)

5. [Pedersen, C. B. *et al.* The iPSYCH2012 case-cohort sample: new directions for unravelling genetic and environmental architectures of severe mental disorders. *Mol. Psychiatry* (2017). doi:](http://paperpile.com/b/78IEi6/gFWu4)[10.1038/mp.2017.196](http://dx.doi.org/10.1038/mp.2017.196)

6. [1000 Genomes Project Consortium *et al.* A global reference for human genetic variation. *Nature* **526,** 68–74 (2015).](http://paperpile.com/b/78IEi6/do7vN)

7. [Manichaikul, A. *et al.* Robust relationship inference in genome-wide association studies. *Bioinformatics* **26,** 2867–2873 (2010).](http://paperpile.com/b/78IEi6/gNuH)

8. [Conomos, M. P., Reiner, A. P., Weir, B. S. & Thornton, T. A. Model-free Estimation of Recent Genetic Relatedness. *Am. J. Hum. Genet.* **98,** 127–148 (2016).](http://paperpile.com/b/78IEi6/9HiM)

9. [Li, J. Z. *et al.* Worldwide human relationships inferred from genome-wide patterns of variation. *Science* **319,** 1100–1104 (2008).](http://paperpile.com/b/78IEi6/0Tmz6)

10. [Schlebusch, C. M. *et al.* Genomic variation in seven Khoe-San groups reveals adaptation and complex African history. *Science* **338,** 374–379 (2012).](http://paperpile.com/b/78IEi6/IpTYq)

11. [May, A. *et al.* Genetic diversity in black South Africans from Soweto. *BMC Genomics* **14,** 644 (2013).](http://paperpile.com/b/78IEi6/8Hwaz)

12. [Patin, E. *et al.* The impact of agricultural emergence on the genetic history of African rainforest hunter-gatherers and agriculturalists. *Nat. Commun.* **5,** 3163 (2014).](http://paperpile.com/b/78IEi6/iN1ki)

13. [Patin, E. *et al.* Dispersals and genetic adaptation of Bantu-speaking populations in Africa and North America. *Science* **356,** 543–546 (2017).](http://paperpile.com/b/78IEi6/53izP)

14. [Triska, P. *et al.* Extensive Admixture and Selective Pressure Across the Sahel Belt. *Genome Biol. Evol.* **7,** 3484–3495 (2015).](http://paperpile.com/b/78IEi6/urKqi)

15. [Fortes-Lima, C. A. *et al.* Genome-wide ancestry and demographic history of African-descendant Maroon communities from French Guiana and Suriname. *In press* (2017).](http://paperpile.com/b/78IEi6/W7FLR)

16. [Reich, D. *et al.* Reconstructing Native American population history. *Nature* **488,** 370–374 (2012).](http://paperpile.com/b/78IEi6/g5N5O)

17. [Mallick, S. *et al.* The Simons Genome Diversity Project: 300 genomes from 142 diverse populations. *Nature* **538,** 201–206 (2016).](http://paperpile.com/b/78IEi6/Twdmy)

18. [Delaneau, O., Zagury, J.-F. & Marchini, J. Improved whole-chromosome phasing for disease and population genetic studies. *Nat. Methods* **10,** 5–6 (2013).](http://paperpile.com/b/78IEi6/xBY0K)

19. [Delaneau, O., Marchini, J., 1000 Genomes Project Consortium & 1000 Genomes Project Consortium. Integrating sequence and array data to create an improved 1000 Genomes Project haplotype reference panel. *Nat. Commun.* **5,** 3934 (2014).](http://paperpile.com/b/78IEi6/t5iCF)

20. [Browning, S. R. *et al.* Local Ancestry Inference in a Large US-Based Hispanic/Latino Study: Hispanic Community Health Study/Study of Latinos (HCHS/SOL). *G3* **6,** 1525–1534 (2016).](http://paperpile.com/b/78IEi6/mwDtj)

21. [Bhatia, G. *et al.* Genome-wide scan of 29,141 African Americans finds no evidence of directional selection since admixture. *Am. J. Hum. Genet.* **95,** 437–444 (2014).](http://paperpile.com/b/78IEi6/F4Q4w)

22. [Jin, W., Wang, S., Wang, H., Jin, L. & Xu, S. Exploring population admixture dynamics via empirical and simulated genome-wide distribution of ancestral chromosomal segments. *Am. J. Hum. Genet.* **91,** 849–862 (2012).](http://paperpile.com/b/78IEi6/lDqPF)

23. [Maples, B. K., Gravel, S., Kenny, E. E. & Bustamante, C. D. RFMix: a discriminative modeling approach for rapid and robust local-ancestry inference. *Am. J. Hum. Genet.* **93,** 278–288 (2013).](http://paperpile.com/b/78IEi6/Qqde8)

24. [Martin, A. R. *et al.* Human Demographic History Impacts Genetic Risk Prediction across Diverse Populations. *Am. J. Hum. Genet.* **100,** 635–649 (2017).](http://paperpile.com/b/78IEi6/ldod)

25. [Moreno-Estrada, A. *et al.* Reconstructing the population genetic history of the Caribbean. *PLoS Genet.* **9,** e1003925 (2013).](http://paperpile.com/b/78IEi6/ReEd)

26. [Fortes-Lima, C. *et al.* Genome-wide Ancestry and Demographic History of African-Descendant Maroon Communities from French Guiana and Suriname. *Am. J. Hum. Genet.* **101,** 725–736 (2017).](http://paperpile.com/b/78IEi6/JHKs)

27. [Bivand, R. & Lewin-Koh, N. maptools: Tools for reading and handling spatial objects. *R package version 0.8* (2013).](http://paperpile.com/b/78IEi6/VgFue)
