# Supplementary material for: Exploring Cuba’s population structure and demographic history using genome-wide data
Source: Sci Rep. 2018 Jul 30;8:11422. doi: 10.1038/s41598-018-29851-3 (PMC6065444; doi:10.1038/s41598-018-29851-3)
Supplement: Supplementary file 2 — All supplementary Tables and supplementary Figures [file 41598_2018_29851_MOESM2_ESM.docx]

**Exploring Cuba’s population structure and demographic history using genome-wide data**

**Cesar Fortes-Lima, Jonas Bybjerg-Grauholm, Lilia Caridad Marin-Padrón, Enrique Javier Gomez-Cabezas, Marie Bækvad-Hansen, Christine Søholm Hansen, Phuong Le, David Michael Hougaard, Paul Verdu, Ole Mors, Esteban J. Parra & Beatriz Marcheco-Teruel**

**Electronic supporting tables and figures**

# Supplementary Tables

**The following tables below can be found in accompanying supplementary Excel file.**

**Supplementary Table S1**. Detailed information of populations included in the Cuba-World SNP dataset.

**Supplementary Table S2**. Admixture membership proportions across Cuban provinces. Table showing average of three- and four-continental ancestry proportions (±standard deviation) in each province, estimated using ADMIXTURE and RFMix (EM=2) analyses.

**Supplementary Table S3**. Average ancestry proportions estimated for short and long ancestry tracts observed in Cuban haploid genomes.

**Supplementary Table S4**. Matrix of IBD lengths within and between Cuban provinces. Table showing the mean size of IBD segments shared between individuals of different provinces (to the right of the diagonal), and the mean length of IBD segments (in Mb) shared per pair of individuals of different provinces (to the left of the diagonal). The length of IBD segments (in Mb) shared per pair of individuals from the same province is indicated in the diagonal. Mean lengths surpassing 6 Mb/pair are labelled in bold.

**Supplementary Table S5**. Autosomal and X-chromosome ancestry proportions in each Cuban province estimated using RFMix (EM=2). Table showing ancestral estimates for both sexes separately, and together based on the sex-biased admixture model. In addition, we indicate the significance obtained in the Wilcoxon signed rank test between autosomal and X-chromosome ancestry proportions.

**Supplementary Table S6**. Detailed information of populations included in the Cuba-Africa SNP dataset.

**Supplementary Table S7**. Detailed information of populations included in the Cuba-America SNP dataset.

**Supplementary Table S8**. Ancestry-specific WC-*F_ST_* values estimated for pairwise populations between Cuban individuals and reference populations with European, African, and Native American ancestry.

**Supplementary Table S9**. Detailed information of populations included in the Cuba-SGDP SNP dataset. Table also showing ancestry-specific WC-*F_ST_* values estimated for pairwise populations between Cuban individuals and reference Native American populations.

**Supplementary Table S10**. Inferred admixture dates estimated using TRACTS analysis for each Cuban region. Dates of the admixture event were calculated assuming 29 years per generation.

# Supplementary Figures


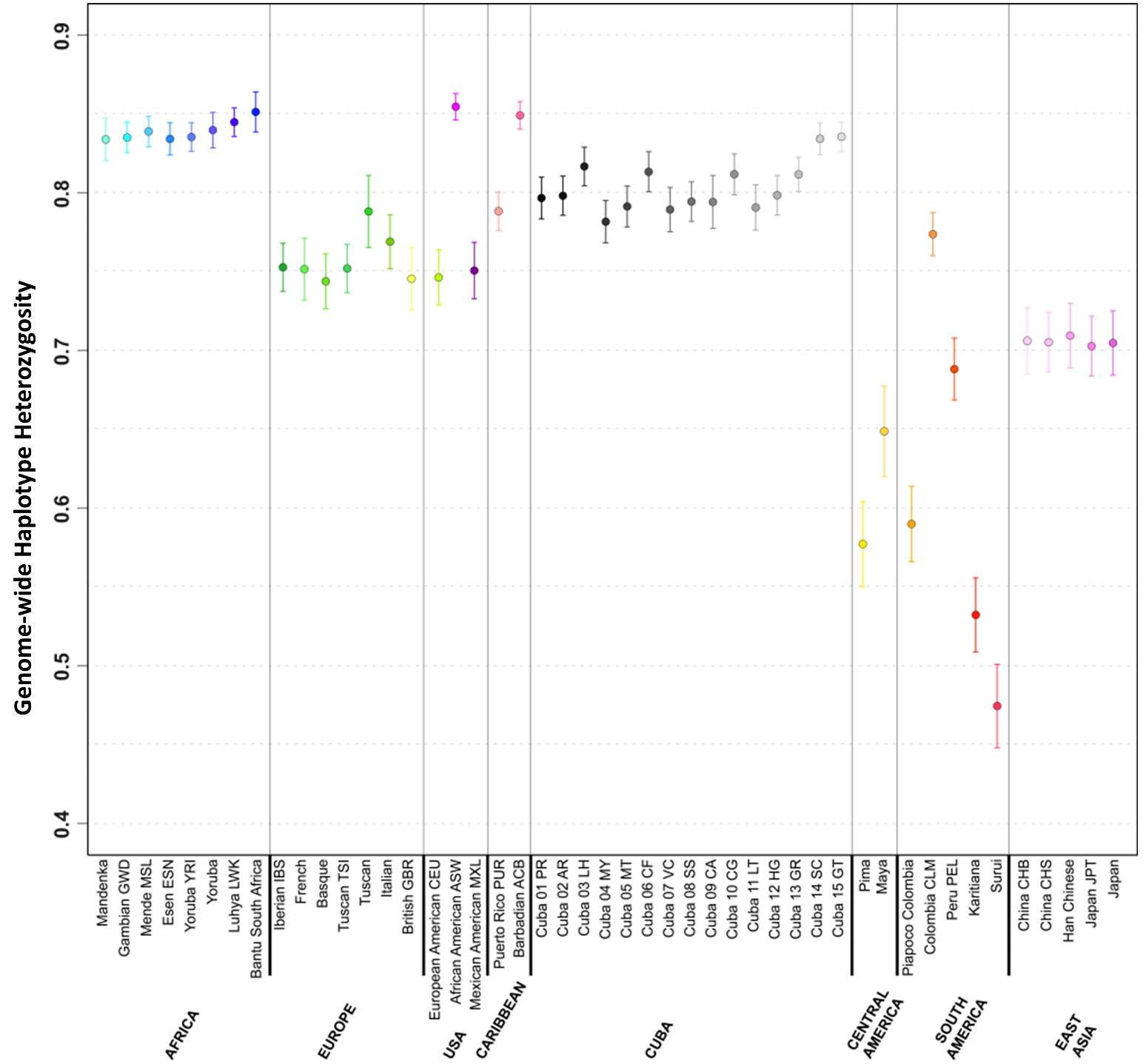


## Supplementary Figure S1. Genome-wide haplotypic heterozygosity in populations included in the Cuban-World dataset (Supplementary Table S1). Dots represents the estimated population-level mean haplotype heterozygosity, and bar lines represents the standard deviation.

**
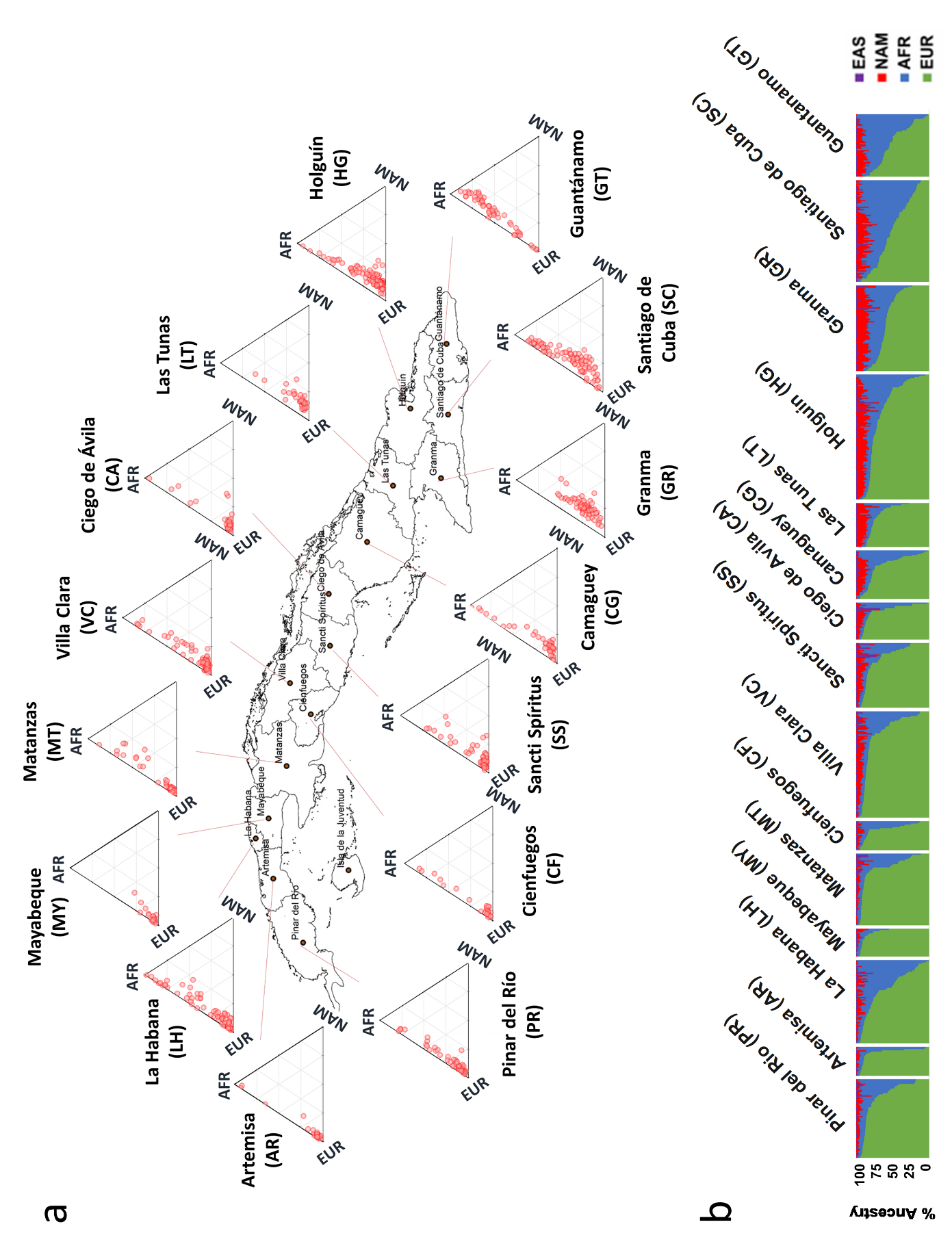
**

**Supplementary Figure S2**. Individual admixture proportions across Cuba obtained using unsupervised ADMIXTURE analysis. (**a**) At K=3, each Cuban individual is represented by a point within the ternary-plots for each Cuban province that in turn shows the autosomal ancestry proportions for European (EUR), African (AFR), and Native American (NAM) ancestry of each individual. (**b**) At K=4, each Cuban individual is represented by a vertical line in each bar-plot that in turn represents the autosomal ancestry proportions for each continental ancestry. The vast majority of the 860 Cuban individuals exhibit significant admixture proportions for European (on average: 71.1% SD=20.2%), African (on average: 20.3% SD=20.4%), and Native American (on average: 6.8% SD=3.6%) ancestry. Only 37 Cuban individuals (4.3% of the Cuban sample) show a small non-residual admixture proportion for the East Asian (EAS) ancestry higher than 5%, hence evidencing the limited contribution of the EAS gene-pool to the Cuban population genetic diversity (on average: 1.8% SD=2.5%).


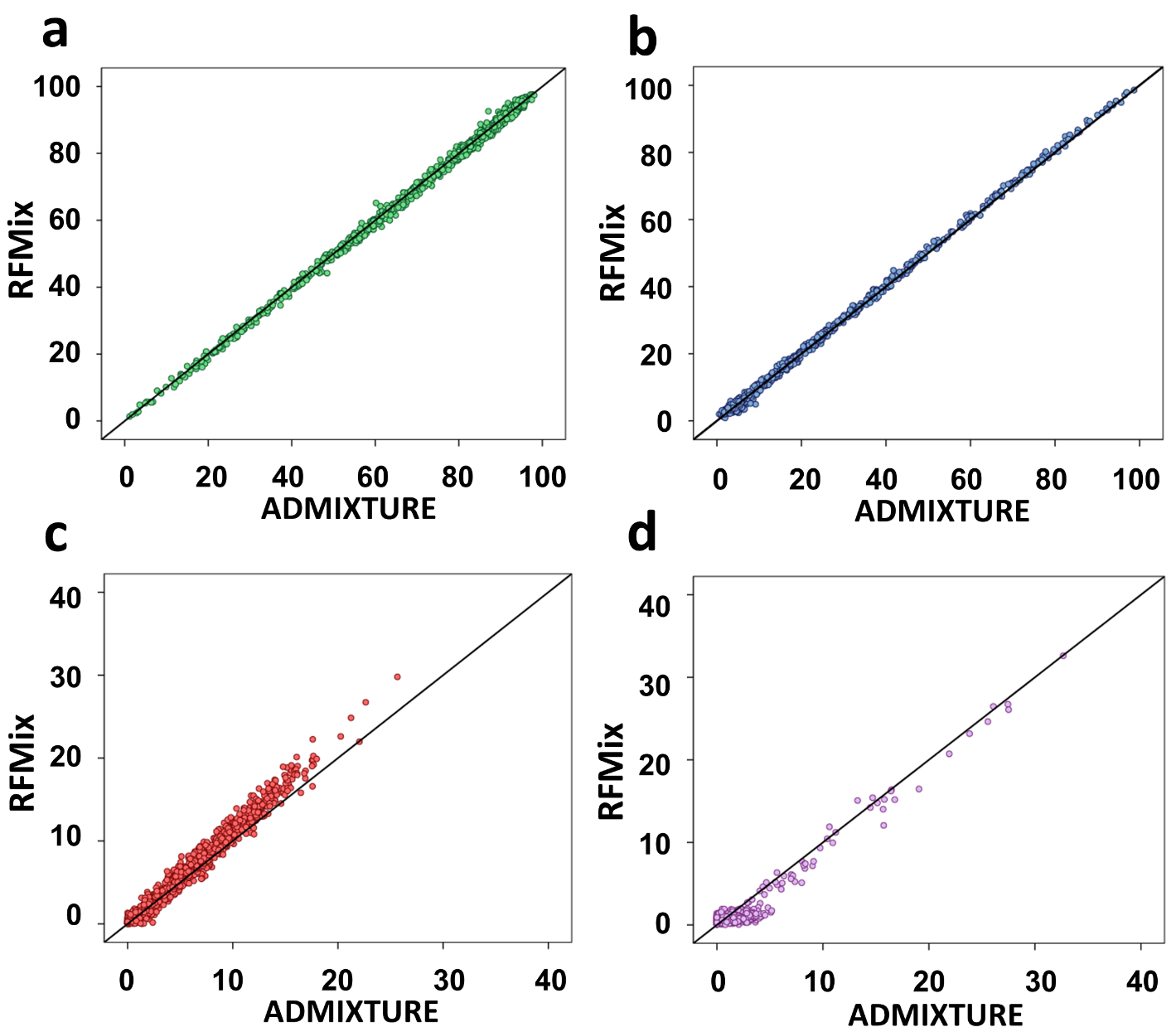


## Supplementary Figure S3. Correlation of individual ancestry proportions obtained in the Cuban population, based on four-way admixture model and using both RFMix (EM=2) and unsupervised ADMIXTURE analysis (K=4). In both analyses, we used 94 samples per continental reference panel and 860 Cuban samples. Figure showing individual ancestry proportions for (a) European, (b) African, (c) Native American, and (d) East Asian ancestries. Spearman’s rho correlation test indicates significant correlation for each continental ancestry: 0.997 (*P*-value<0.001) for EUR ancestry, 0.986 (*P*-value<0.001) for AFR ancestry, 0.986 (*P*-value<0.001) for NAM ancestry, and 0.537 (*P*-value<0.001) for EAS ancestry.


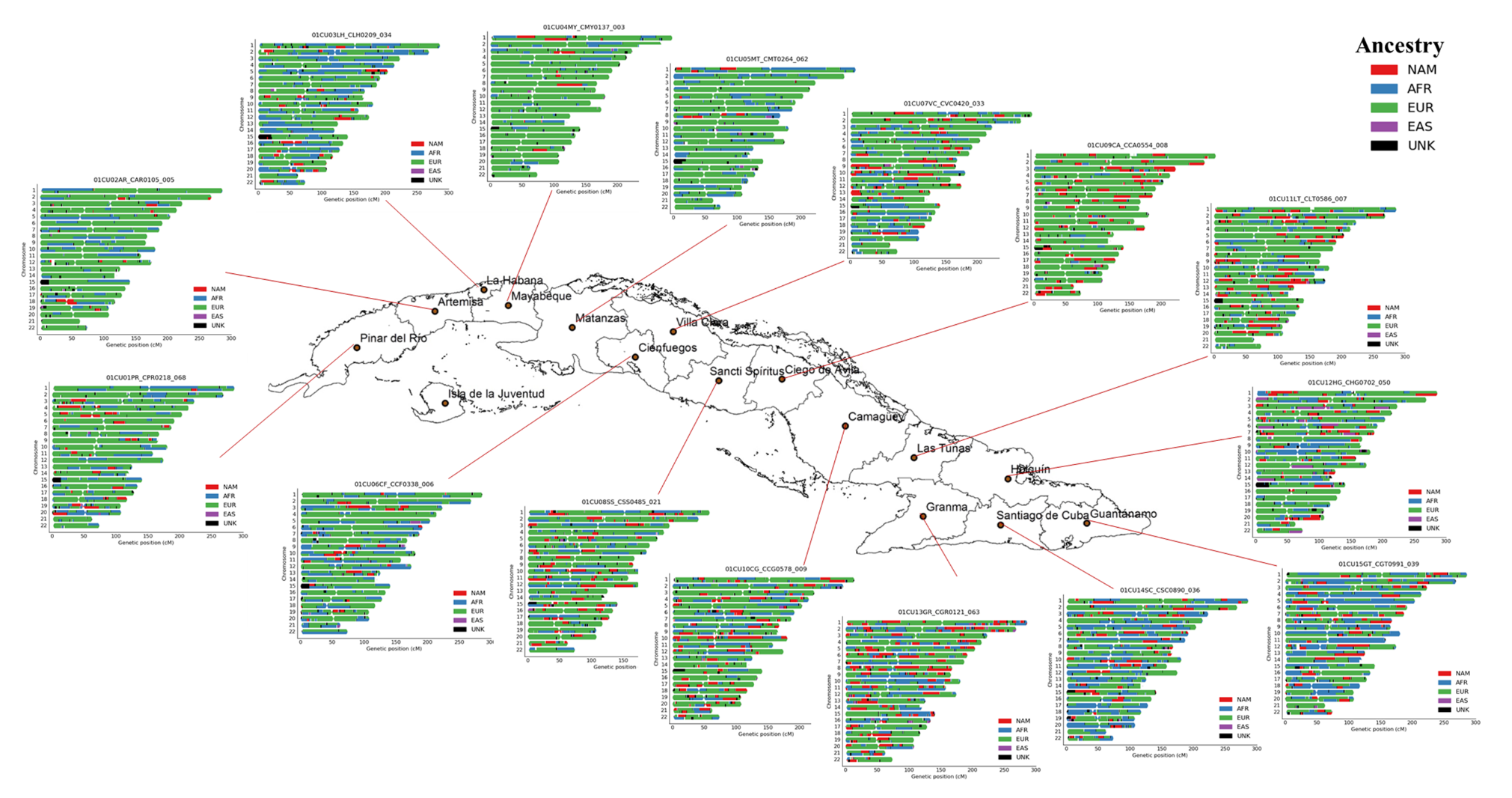


## Supplementary Figure S4. Cuban individual haploid genomes estimated using RFMix (EM=2) analysis based on a four-way admixture model. Figure showing the individual who has ancestral proportions that are similar to the average in their respective Cuban province (see Supplementary Table S2).


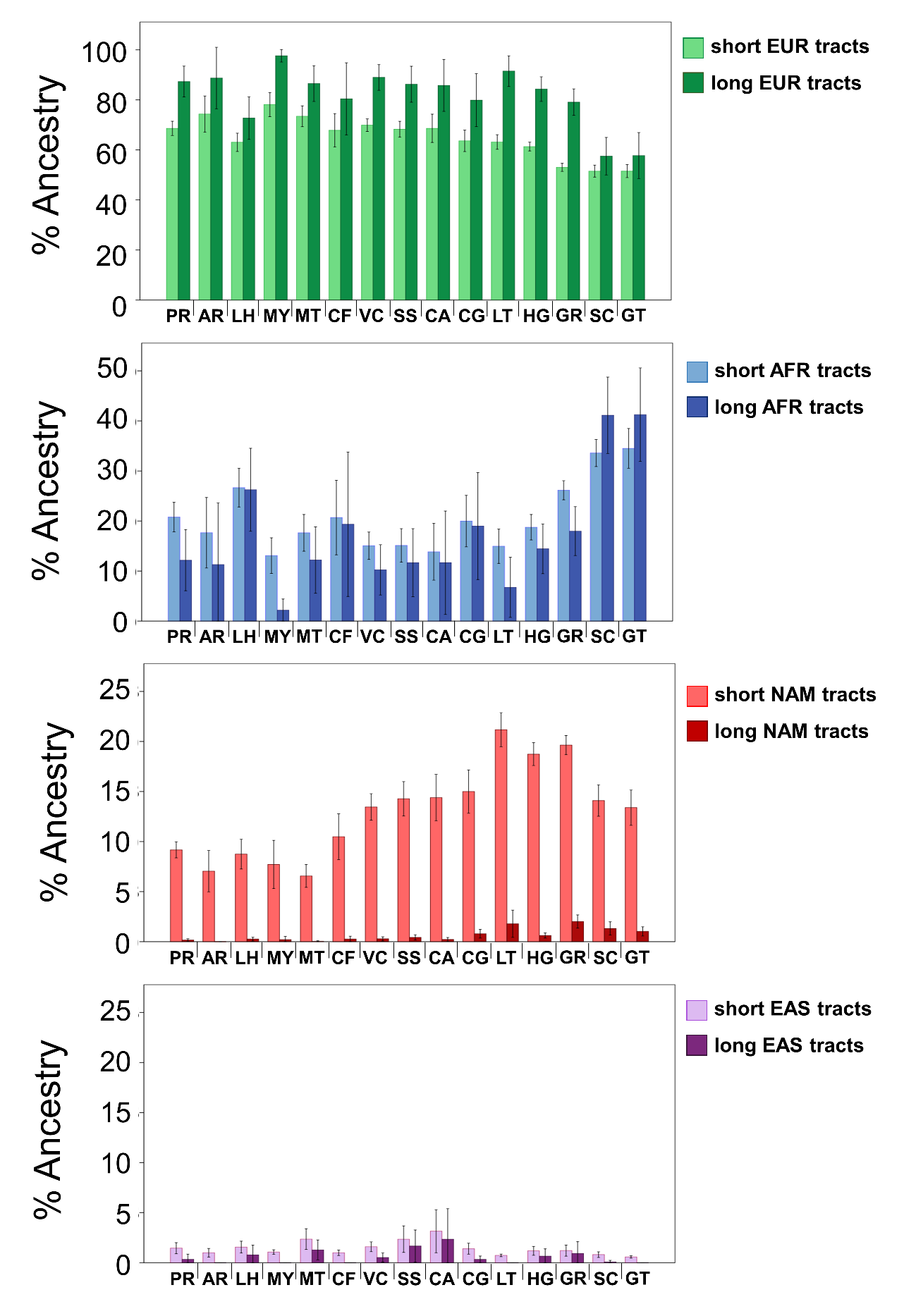


## Supplementary Figure S5. Average ancestry proportions in short (between >5 and ≤50cM) and long (>50cM) ancestry tracts. Figure showing averages estimated in each Cuban province using RFMix (EM=2) and based on a four-way admixture model for European (EUR), African (AFR), Native American (NAM), and East Asian (EAS) ancestry (Supplementary Table S3). Bar-plots were plotted with respective confidence intervals (95% CI).


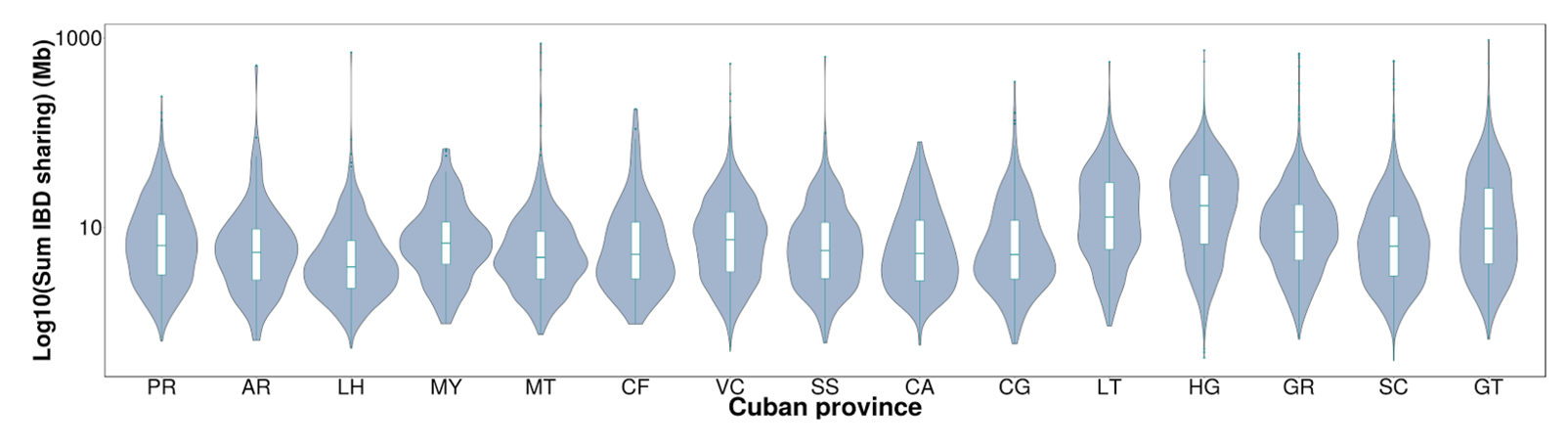


## Supplementary Figure S6. Distribution of the sum of identity-by-descent (IBD) sharing between pairs of individuals within Cuban provinces. The violin plot shows the variation present in each province. Las Tunas and Holguin have the highest averages in Cuba (17.0 and 18.5 Mb/pair, respectively), in contrast with La Havana that has the lowest average in Cuba (1.55 Mb/pair).


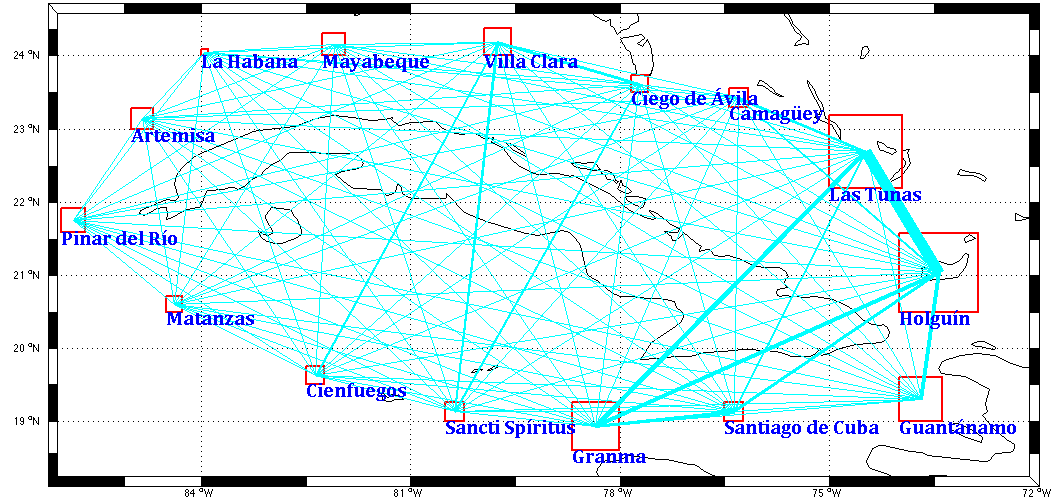


## Supplementary Figure S7. Average cumulative length of the IBD segments shared by individuals between (lines) and within (squares) Cuban provinces. The thickness of the lines is proportional to the average total length (in Mb) of IBD segments shared per pair of individuals. Note the thicker line connecting the Eastern provinces of Las Tunas and Holguin. The size of the red squares is proportional to the average total length of IBD segments shared by individuals within each Cuban province. Note the larger size of the squares corresponding to the provinces of Holguin, Las Tunas, Gramma, and Guantanamo.


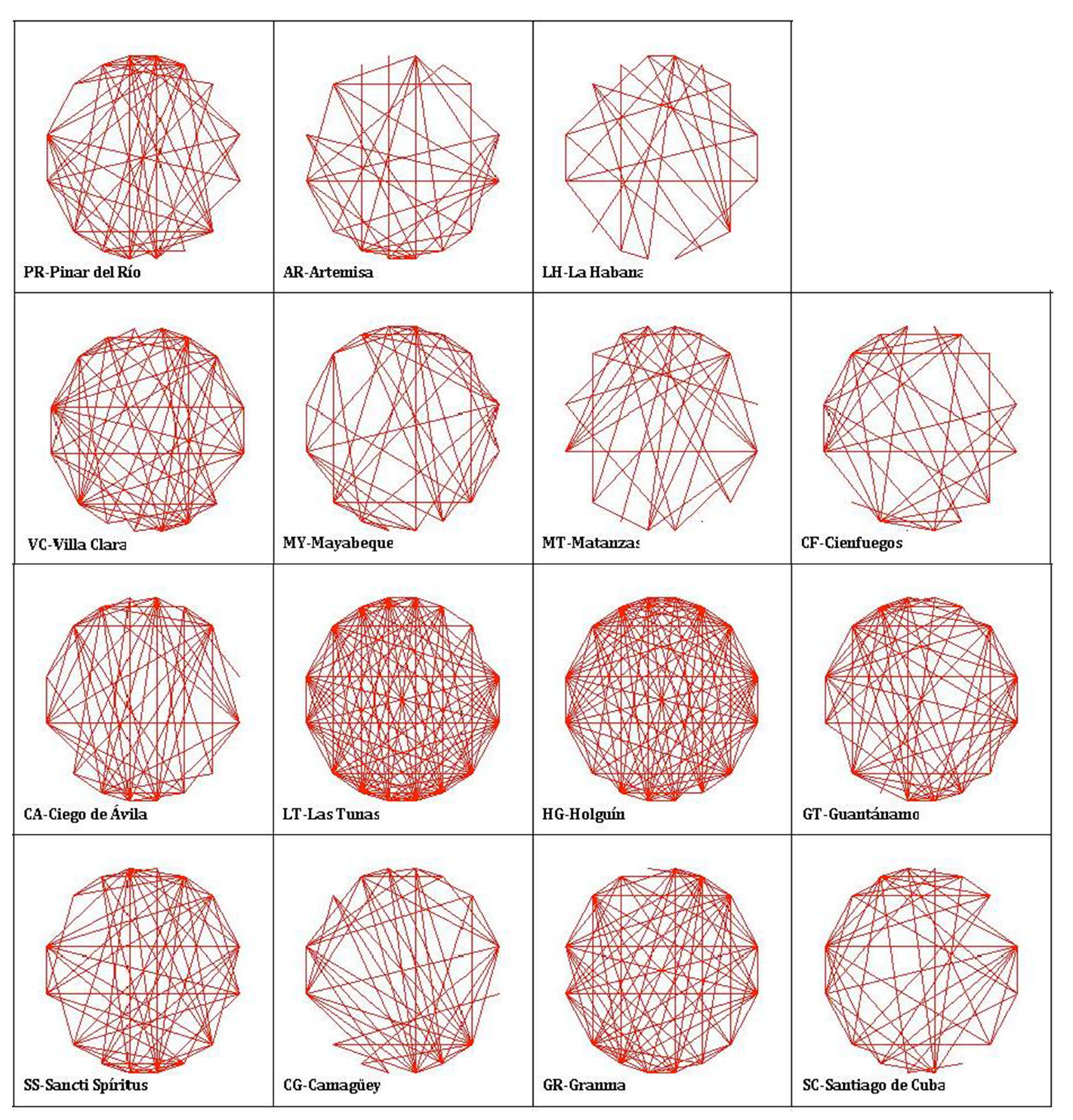


## Supplementary Figure S8. Graphical representation of IBD segments shared between 16 randomly selected individuals for each of the Cuban provinces. Note the denser network in the IBD sharing patterns in the Eastern provinces of Las Tunas and Holguin.


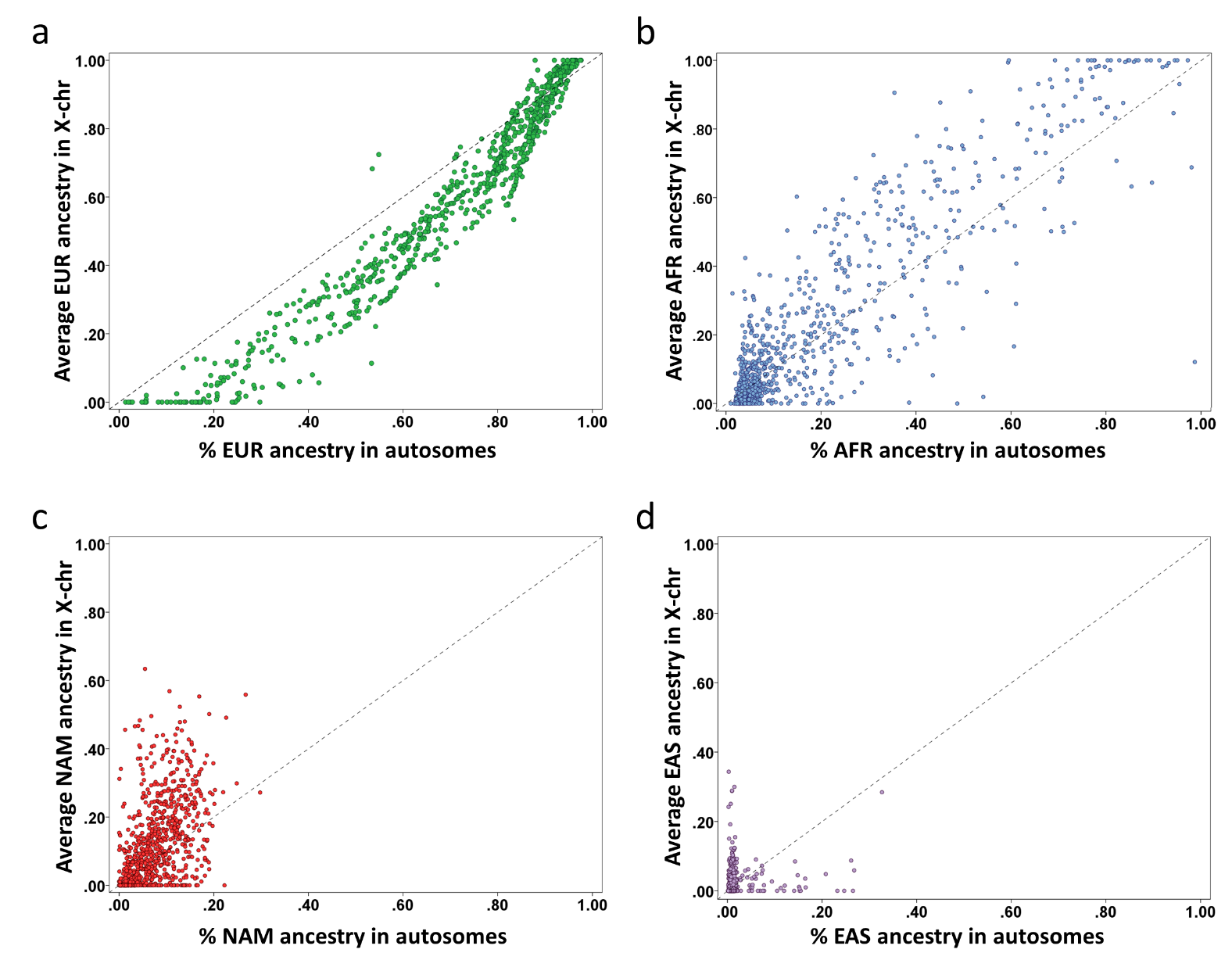


## Supplementary Figure S9. Scatter plot for individual ancestry estimates based on the Cuban dataset for autosomes and X-chromosome using RFMix (EM=2). For each Cuban individual, figure showing estimates for (a) European (EUR), (b) African (AFR), (c) Native American (NAM), (d) East Asian (EAS) ancestry.


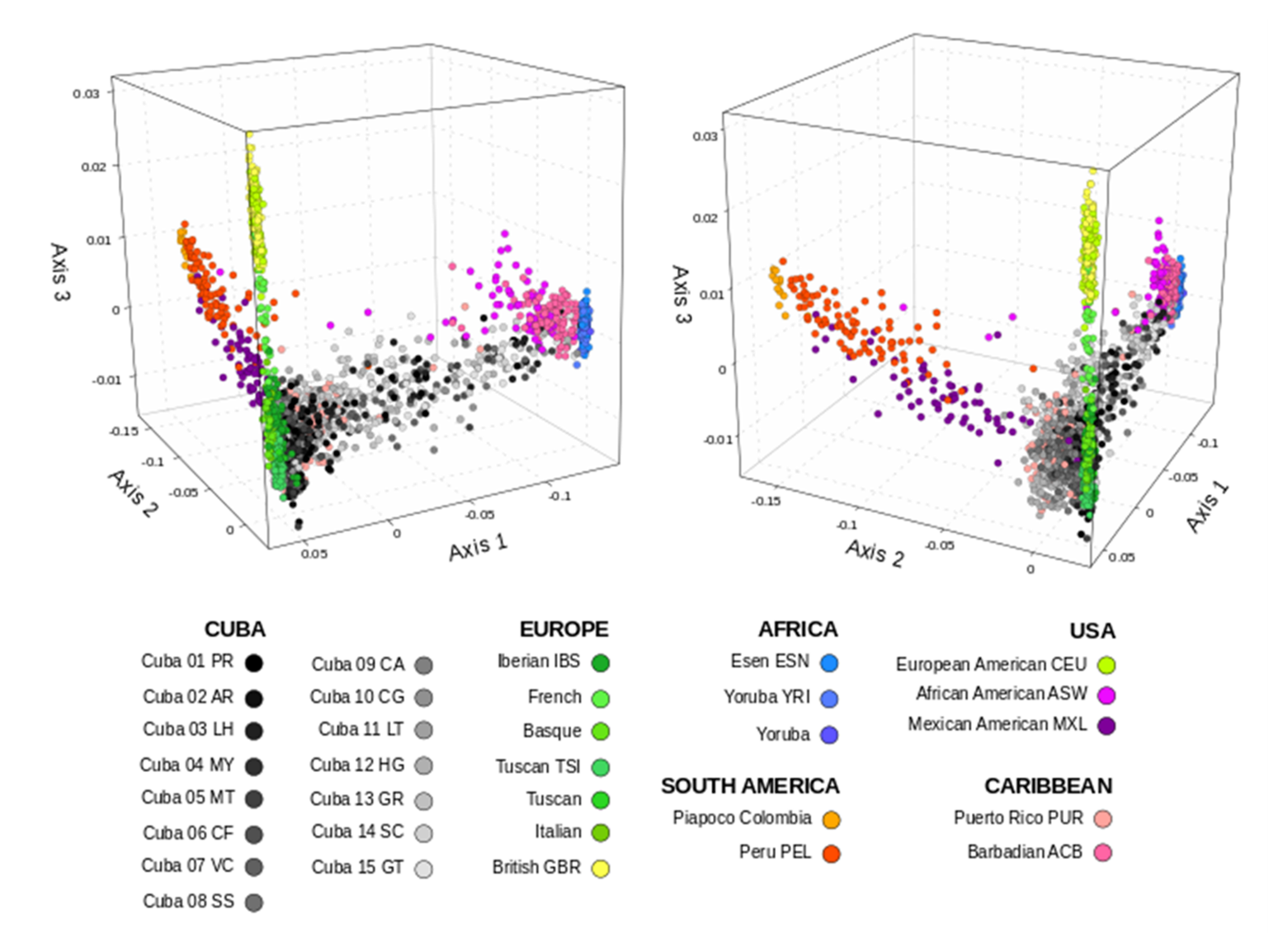


## Supplementary Figure S10. Procrustes-transformed MDS plots focused on European ancestry. Plots included individuals from the fifteen Cuban provinces (black and grey dots) and selected worldwide populations from Europe, Africa, and America included in the Cuba-World dataset (Supplementary Table S1). Cuban individuals present a trajectory to Southern European populations. Hispanic/Latino populations show similar genetic affinities than the Cuban population.


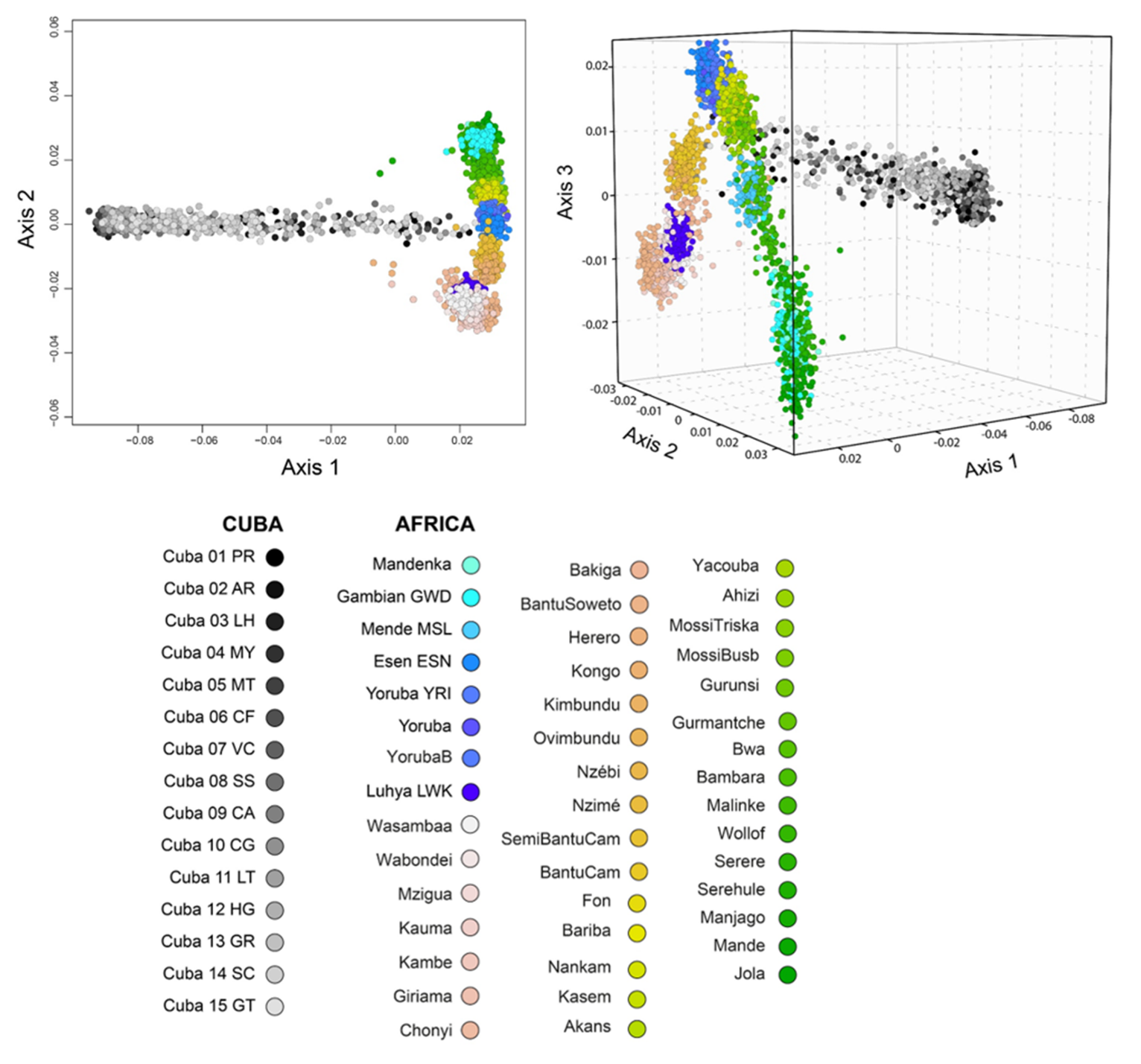


## Supplementary Figure S11. Procrustes-transformed MDS plots focused on 860 Cuban individuals and individuals from 45 sub-Saharan African populations included in the Cuba-Africa dataset (Supplementary Table S6).


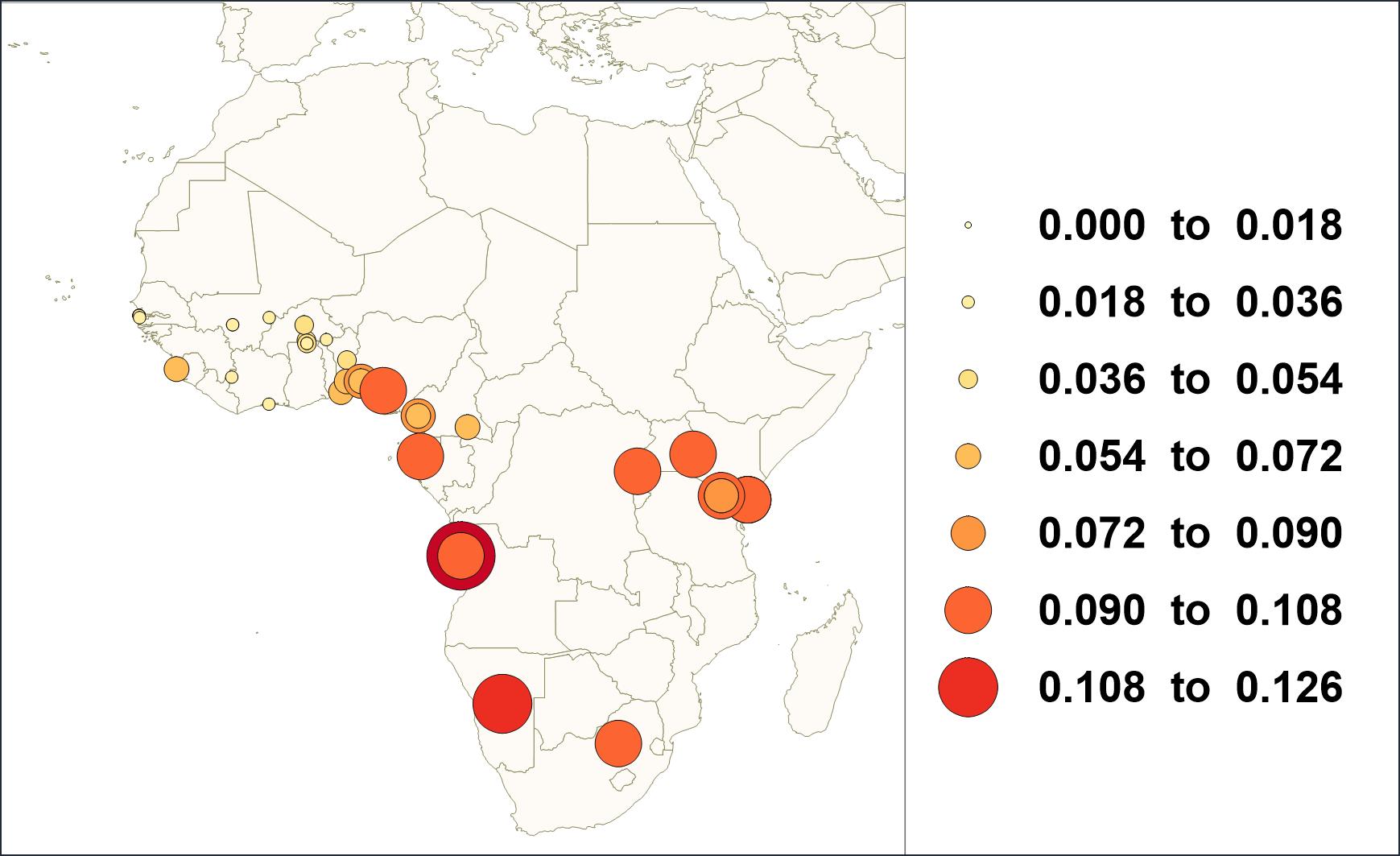


## Supplementary Figure S12. Average cumulative length of the IBD segments shared (in Mb) between the Cuban population and sub-Saharan African populations (Supplementary Table S6).


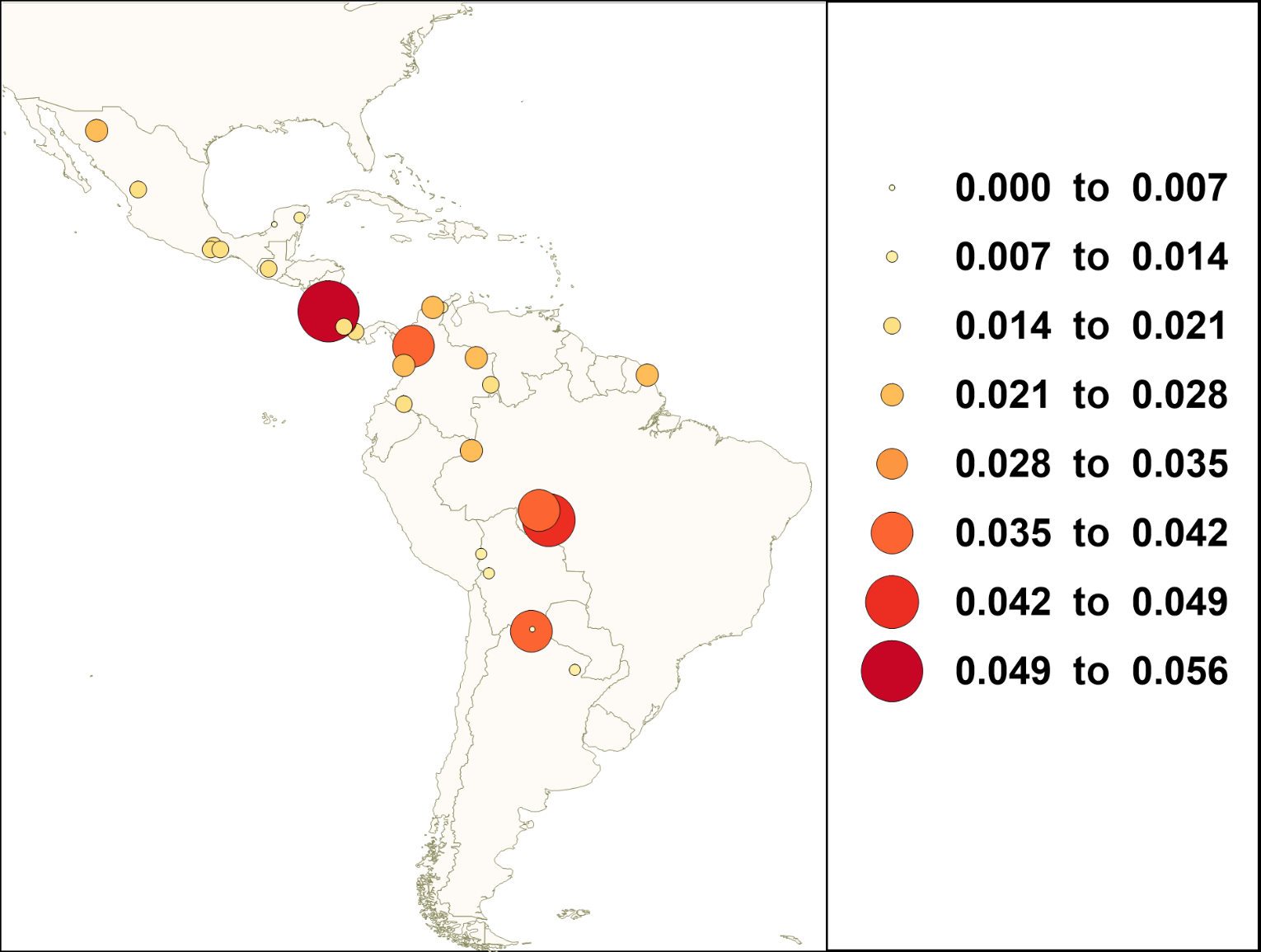


## Supplementary Figure S13. Average cumulative length of the IBD segments (in Mb) shared between the Cuban population and Native American populations across Latin America (Supplementary Table S7).


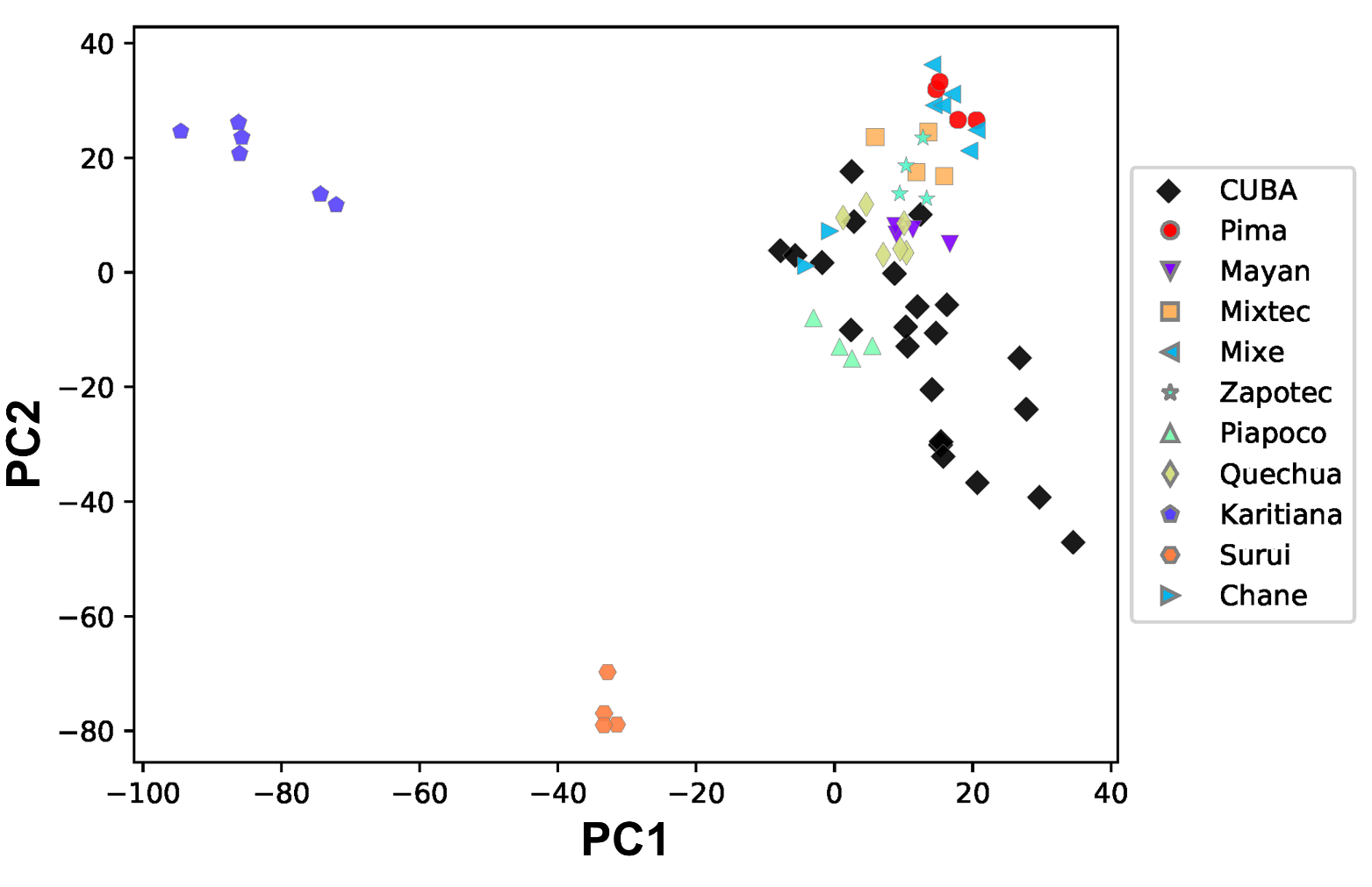


## Supplementary Figure S14. Genetic structure across non-masked reference Native American populations and masked Cuban haploid genomes. Figure showing the MDS-based ASPCA obtained for Native American ancestry using Cuban samples and Native American samples included in the SGDP (Supplementary Table S9). Cuban individuals with more than 15% Native American ancestry based on RFMix (EM=2) analysis were included in the analysis,


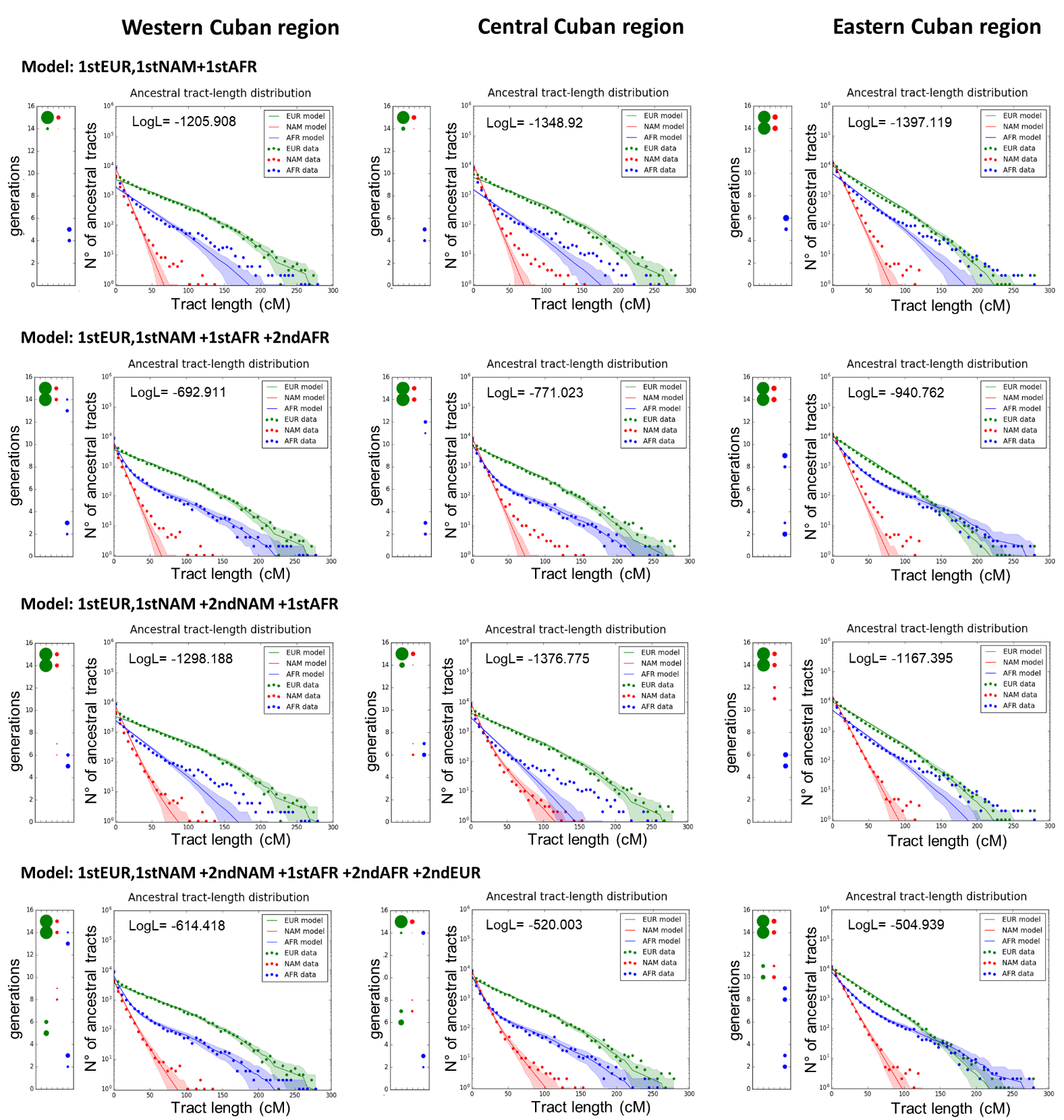


## Supplementary Figure S15. TRACTS analysis testing four different migration models in the three Cuba regions. Figure showing tested demographic scenarios for best fitting the observed data in each Cuban region, based on the strength of the log-likelihood (LogL) of the composite demographic model. For each historical region, we tested five admixture models with different admixture pulses for European (EUR), Native American (NAM), and African (AFR) ancestry.

## Scatter data points represent the observed distribution of Cuban ancestry tracts, and solid-coloured lines represent the distribution from the model, with shaded areas indicating 68.3% confidence intervals for the predicted migration model. The best-fitting model for each region is depicted in Figure 5.


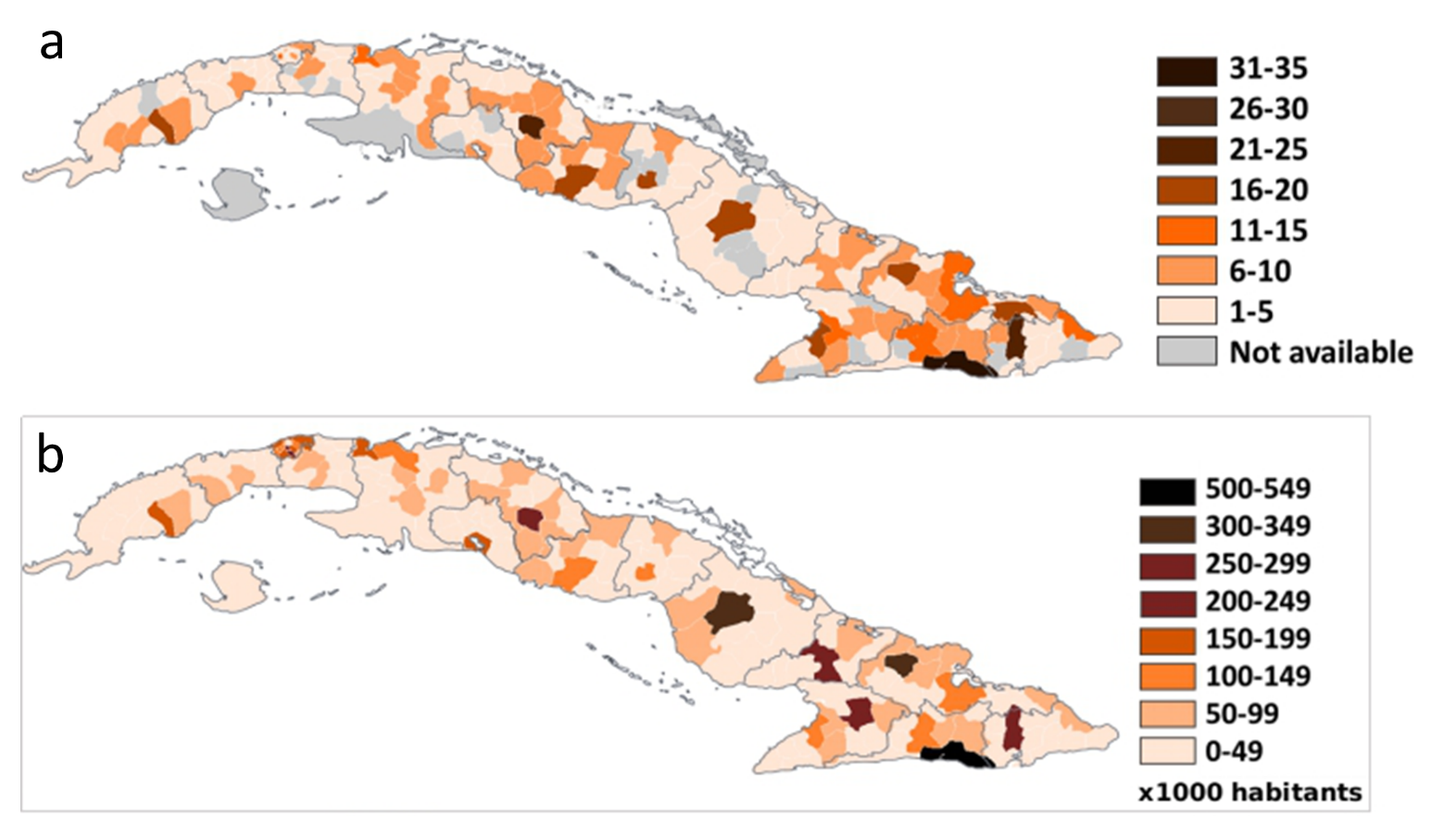


## Supplementary Figure S16. Geographical locations of Cuban individuals analysed in the present study and in the last Cuban census. (a) Figure shows the number of samples collected in that area based on the administrative division of Cuban territory by provinces and municipalities. (b) The population density of each Cuban province and each Cuban municipality is concordant with the 2012 census of the Cuban population.


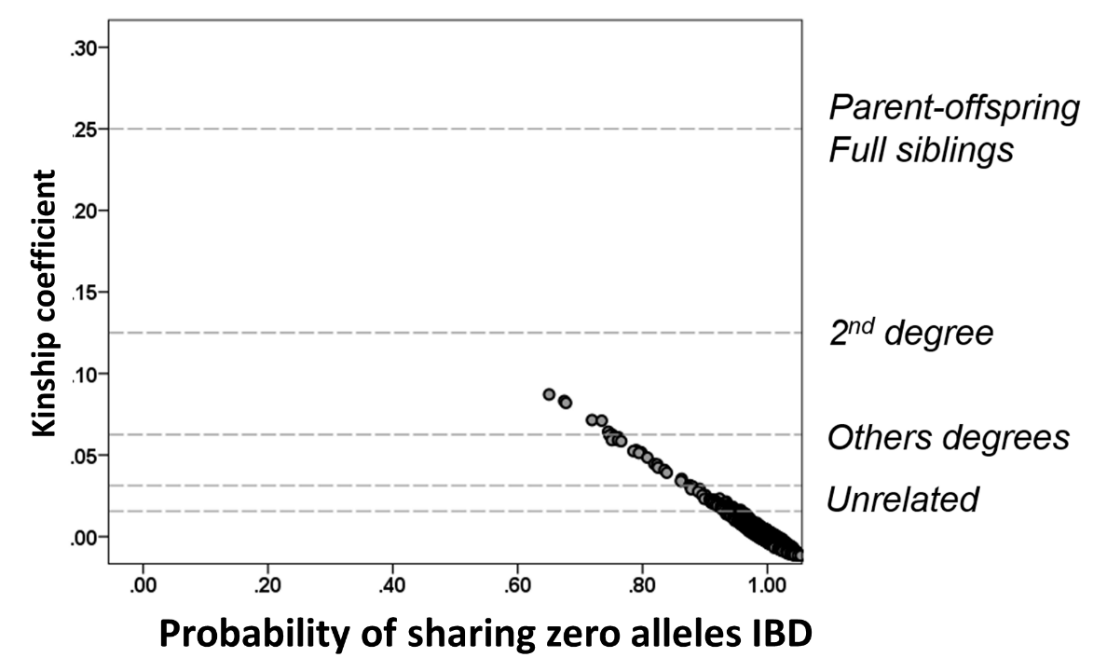


## Supplementary Figure S17. Cuban dataset included 864 unrelated individuals. Figure showing the low relatedness probabilities between the Cuban samples according to the kinship coefficient estimated using PC-Relate, and no first-degree or second-degree relatives were found.


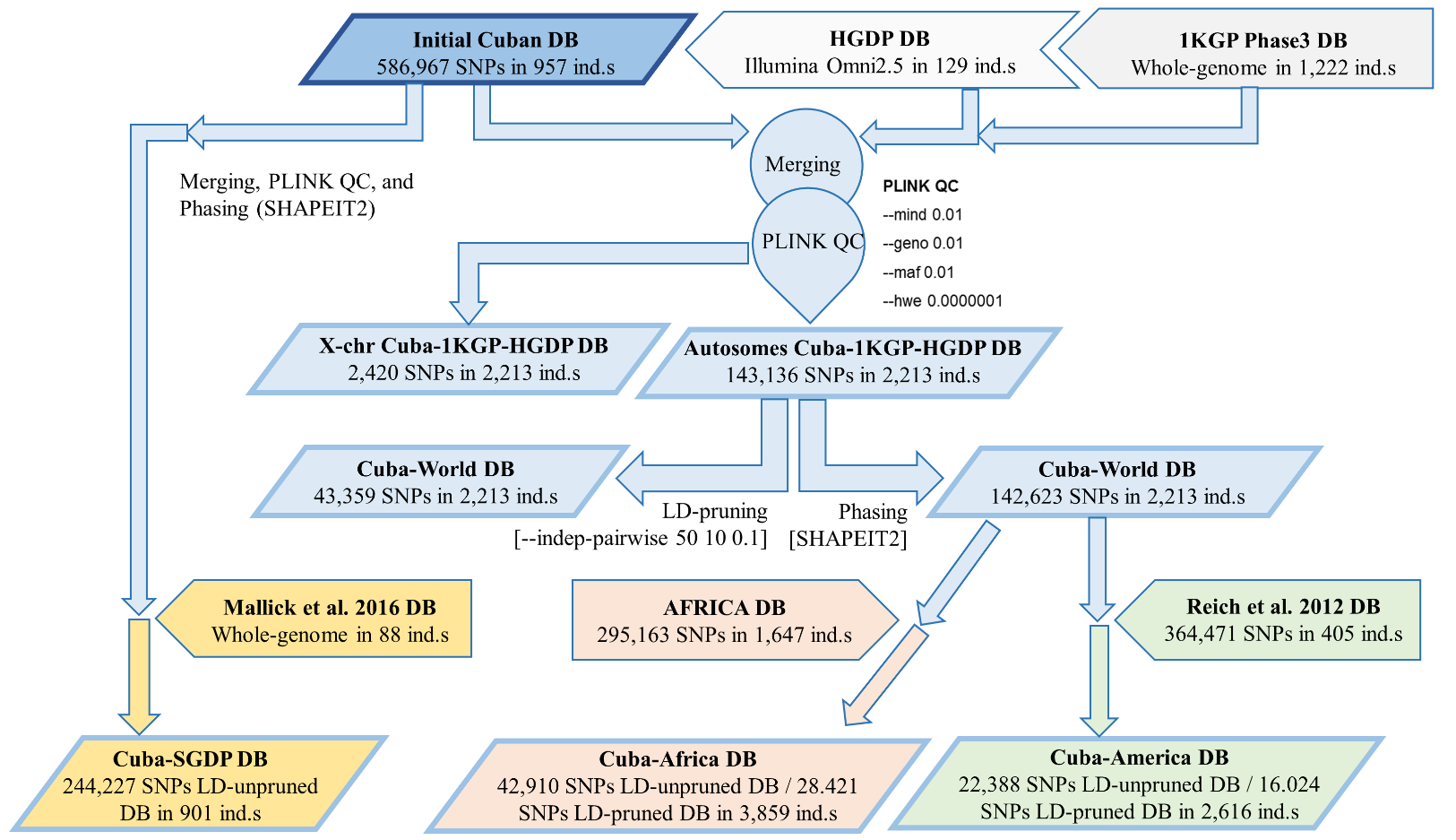


## Supplementary Figure S18. Flowchart summarizing the construction of the assembled genome-wide SNP datasets that were used in the distinct stages of our analyses of the Cuban population.
